# Chromosomal instability causes sensitivity to protein folding stress and ATP depletion

**DOI:** 10.1242/bio.038000

**Published:** 2018-10-15

**Authors:** Mahwish Khan, Zeeshan Shaukat, Robert Saint, Stephen L. Gregory

**Affiliations:** 1 Department of Genetics, University of Adelaide, Adelaide 5006, Australia; 2 College of Medicine and Public Health, Flinders University, Adelaide 5042, Australia

**Keywords:** Endoplasmic reticulum, Protein aggregation, *Drosophila*, Genomic instability, Reactive oxygen species (ROS)

## Abstract

Aneuploidy _­_– having an unbalanced genome – is poorly tolerated at the cellular and organismal level. It gives rise to proteotoxic stress as well as a stereotypical oxidative shift which makes these cells sensitive to internal and environmental stresses. Using *Drosophila* as a model, we found that protein folding stress is exacerbated by redox stress that occurs in response to ongoing changes to ploidy (chromosomal instability, CIN). We also found that if *de novo* nucleotide synthesis is blocked, CIN cells are dependent on a high level of lysosome function to survive. Depletion of adenosine monophosphate (AMP) synthesis enzymes led to DNA damage in CIN cells, which showed elevated activity of the DNA repair enzyme activated poly(ADP ribose) polymerase (PARP). PARP activation causes depletion of its substrate, nicotinamide adenine dinucleotide (NAD+) and subsequent loss of Adenosine Tri-Phosphate (ATP), and we found that adding ATP or nicotinamide (a precursor in the synthesis of NAD+) could rescue the observed phenotypes. These findings provide ways to interpret, target and exploit aneuploidy, which has the potential to offer tumour-specific therapies.

## Abstract (precis)

**Summary:** Cells that gain or lose chromosomes during cell division are shown to be sensitive to ATP levels and protein folding stress.

## INTRODUCTION

Aneuploidy is known to have detrimental effects on most cells, including reduced proliferation and elevated redox stress (Sheltzer et al., 2017; Siegel and Amon, 2012). The concept of ‘unbalanced’ mutations giving a phenotype when single chromosomes are gained or lost is now almost a century old (Blakeslee et al., 1920). In yeast, changes in gene dosage brought about by aneuploidy have been shown to generate an excess of some proteins, and increase the burden on the protein degradation apparatus (Dephoure et al., 2014). The additional demand for Adenosine Tri-Phosphate (ATP) to synthesize, fold and turn over these excess proteins has been proposed to cause increased mitochondrial activity and the production of reactive oxygen species (ROS). ROS could also be produced from a stressed endoplasmic reticulum if there were protein folding problems (Malhotra and Kaufman, 2007) but, in disomal yeast at least, activation of the unfolded protein response was not detected (Oromendia et al., 2012). Nonetheless, protein aggregation was increased by aneuploidy and, in vertebrate cells, aneuploidy has been linked with increased autophagy and overloading of chaperones such as HSP90 (Donnelly et al., 2014; Santaguida et al., 2015). Consequently, it seems likely that aneuploidy imposes a chronic strain on protein homeostasis, with the exact phenotype depending partly on the particular gene dosage changes in each aneuploid cell, but also on the type of cell. With the whole protein production and turnover system under strain, cell types are likely to differ in what breaks first – limiting levels of antioxidants, chaperones, ATP or lysosome function have all been implicated in different cell types.

The unfolded protein response is typically activated in situations where the endoplasmic reticulum (ER) is unable to manage the load of protein folding that is required, and there is some evidence, at least in vertebrate cells, that it can be induced by aneuploidy. Studies differ as to whether simple tetraploidy is (Senovilla et al., 2012) or is not (Ohashi et al., 2015) enough to trigger robust activation of the unfolded protein response. Furthermore, it is not clear whether aberrant protein levels are the primary cause, because aneuploid cells typically also have an aberrant metabolism that generates ROS and redox stress. Protein folding is a highly redox sensitive process, so aneuploidy may both increase the load of proteins to be folded and impair the ability to process them.

Nucleotide depletion is another homeostatic imbalance that affects aneuploid cells (Bester et al., 2011). Insufficient nucleotides during S-phase lead to replication stress, which has been strongly linked to the induction of chromosomal instability (CIN) that produces highly aneuploid cells. Several mechanisms linking replication stress and CIN have been demonstrated, including increased double stranded DNA breaks, increased anaphase bridges, segregation errors and production of ROS (Burrell et al., 2013; Chan et al., 2009; Marchetti et al., 2006; Minocherhomji et al., 2015). Consequently, we expect that aneuploid cells will be sensitive to perturbation of nucleotide levels, which may increase aneuploidy beyond the cell's threshold of tolerance.

We wished to understand the causal relationships between aneuploidy and these failures of homeostasis because chromosomal instability and its consequent aneuploidy is a common, tumour-specific phenotype that offers the prospect of similarly tumour-specific therapies. This will require the identification of features of aneuploid cells that are invariant, regardless of which DNA has been gained or lost. In this paper we describe a common phenotype of protein folding stress in genetically diverse aneuploid cells using a *Drosophila* model for chromosomal instability. We find that the protein folding defects are caused by redox stress and contribute to that stress. We have tested nucleotide depletion and find that it can be tolerated by aneuploid cells without causing redox stress, but this tolerance requires high levels of lysosome function. Finally, we find that CIN cells are sensitive to decreased ATP synthesis and have activated poly(ADP ribose) polymerase (PARP), which leaves these cells deficient in NAD+ and ATP.

## RESULTS

### CIN causes ER stress and oxidative stress in *Drosophila*

**Fig. 1.**
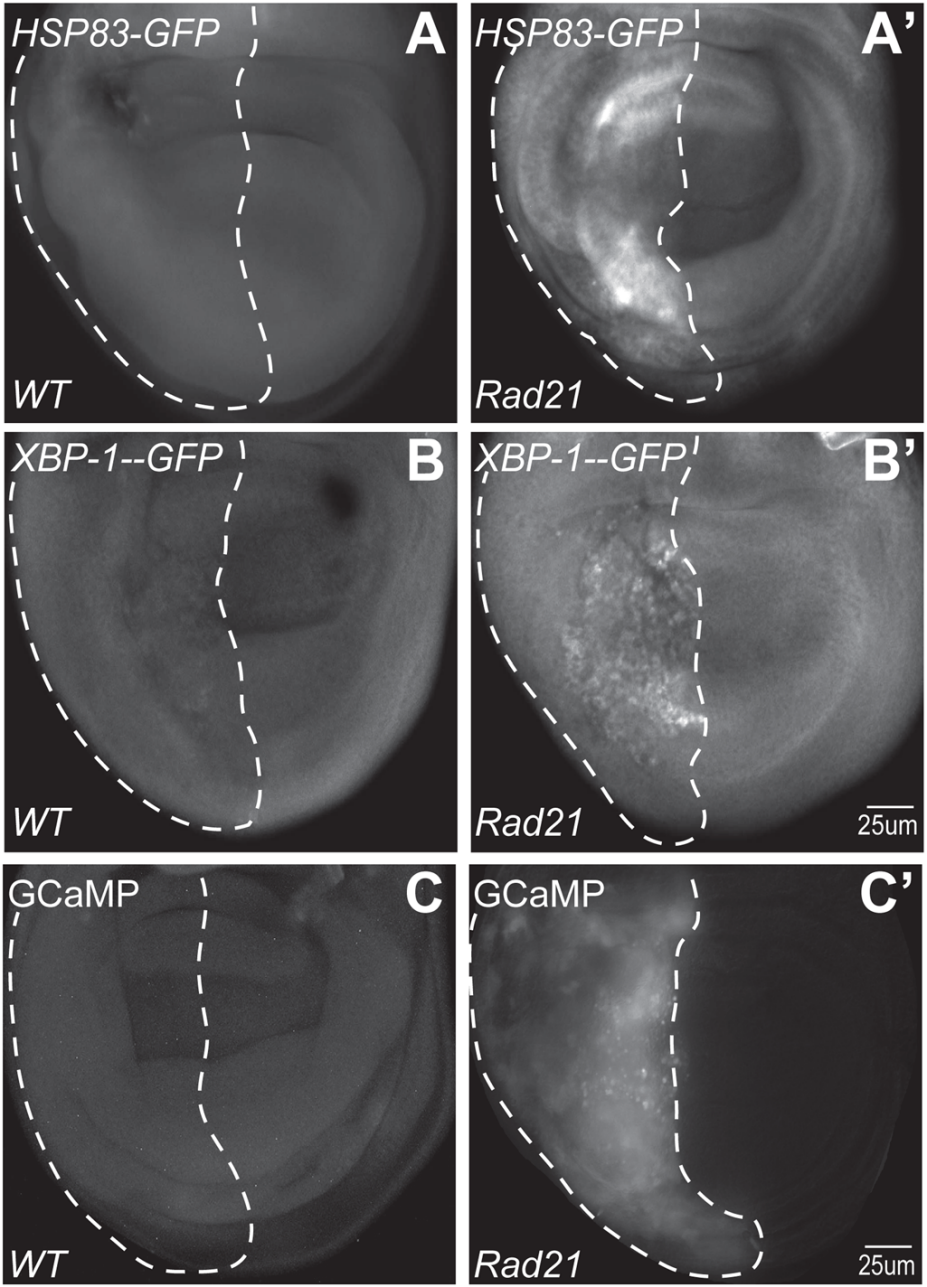
**CIN cells are under ER stress.** ER stress was detected by the levels of chaperones (HSP83-GFP expression, A,A′), the activation of XBP1 by alternative splicing (XBP1-GFP expression, B,B′) and measuring intracellular Ca^++^ release (GCaMP3 signal, C,C′) in CIN (A′,B′,C′) versus normal (A,B,C) wing imaginal tissue. The dotted lines show the posterior compartment of a representative wing disc pouch region from each genotype. (A,B,C) Control wing discs show background expression of HSP-83-GFP, XBP1-GFP and GCaMP3 in non-CIN cells. (A′,B′) Increased GFP levels which indicate elevated levels of chaperone (Hsp83-GFP) and XBP1-GFP were observed when CIN was induced by depletion of Rad21 (UAS-Rad21^RNAi^, UAS-Dicer2). (C′) Increased GCaMP signal was observed in CIN cells relative to wild-type controls (C). These data are quantified in Fig. S1.

Upregulation of protein chaperones has been observed in aneuploid cells from budding yeast, mouse and human (Pfau and Amon, 2012; Sheltzer et al., 2012). To determine whether cells in which aneuploidy was induced by chromosomal instability (CIN) were under protein folding stress, we tested levels of the HSP70 family. We observed an increase in chaperone levels (HSP 83) in cells in which CIN had been induced by Rad21 depletion relative to wild-type controls (Fig. 1A). In this paper we use two models for induced CIN: depletion of the cohesin Rad21, which makes 46% of metaphases aneuploid, typically with gain or loss of a whole chromosome, and causes ROS and cell death (Liu et al., 2015). Alternatively we use depletion of the spindle checkpoint protein Mad2, which causes bridges or lagging chromosomes in approximately 25% of anaphases and gives very little cell death (Shaukat et al., 2012). We typically use high CIN (Rad21 depletion) where we are measuring strong CIN phenotypes and low CIN (Mad2 depletion) where we are testing for genetic enhancement of mild CIN effects. The transcription factor X-box-binding protein 1 (XBP1) is required for the activation of several unfolded protein response genes that control ER protein folding, intracellular trafficking and ER membrane expansion in response to ER stress (Bravo et al., 2013). To observe ER stress in CIN cells, we used recombinant UAS-XBP1-GFP which only produces GFP when its mRNA is appropriately spliced in response to ER stress (Ryoo, 2015; Samali et al., 2010). We found that GFP was increased, indicating the presence of ER stress in CIN cells (Fig. 1B′; Fig. S1). We have previously observed that CIN cells have elevated levels of ROS and mitochondrial dysfunction (Shaukat et al., 2015), so we wished to test whether that might be caused by release of Ca^++^ ions from the stressed ER. The ER is a principal cytoplasmic store for Ca^++^, which can be released in response to protein folding stress, potentially causing loss of mitochondrial integrity and ROS generation (Pinton et al., 2008). We observed an increase in GCaMP3 signal (Tian et al., 2009), which indicates increased Ca^++^ release in CIN cells relative to controls (Fig. 1C).

### CIN affects protein aggregation in *Drosophila*

**Fig. 2.**
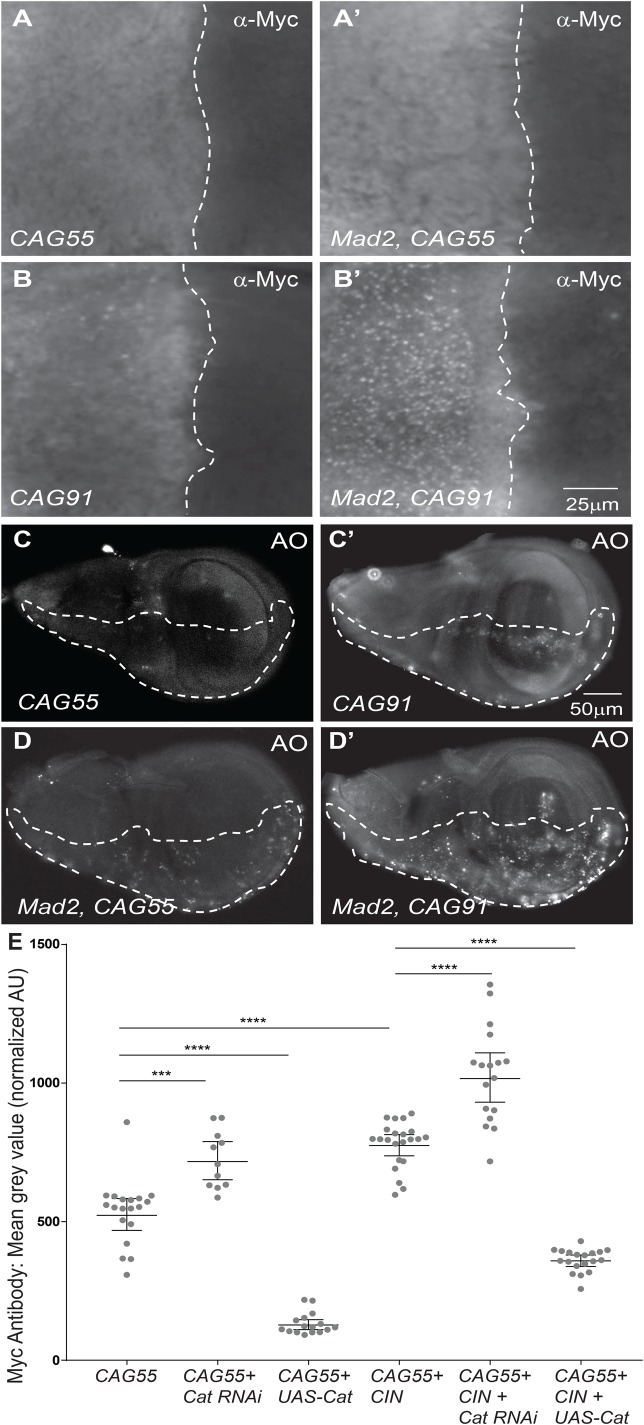
**CIN increases protein folding stress via reactive oxygen species.** Increased aggregation of hard-to-fold polyQ protein (myc-tagged CAG91 or CAG55) was observed in CIN cells. (A,B) Images show the anterior/posterior boundary of a representative wing disc from each genotype. Right of the dotted line is the *engrailed*-Gal4 expressing region in which myc-tagged poly-CAG peptides were expressed. (A) In cells expressing a shorter polyQ peptide (CAG55), aggregation was not observed, and mild CIN induction by Mad2 depletion had little, if any, effect on this (A′). (B) Some aggregation was observed of the hard-to-fold longer polyQ peptide CAG91 in otherwise wild-type cells, which was greatly increased by the induction of CIN (*mad2*^RNAi^, B′). (C,C′) Third instar imaginal discs were stained for Acridine Orange (AO) to indicate vesicle acidification, a common precursor to cell death. The dashed line indicates the posterior compartment expressing CAG55 or CAG91 peptides, while the unmarked anterior compartment was wild type in every disc. (D,D′) Imaginal discs in which CAG55 and CAG91 peptides were expressed in CIN cells (*mad2*^RNAi^), gave rise to high levels of AO staining compared to the non-CIN controls. (E) Quantification of polyQ peptide aggregation shows that ROS significantly contributes to the proteotoxic stress generated in CIN cells. Aggregation of myc-tagged CAG55 increased when co-expressed with catalase-RNAi, while aggregation decreased when catalase was overexpressed (UAS-Catalase). Overexpression of catalase was able to strongly rescue the aggregation seen in CIN cells. The signal was normalized by subtracting the signal from the wild-type anterior compartment of each disc. Error bars indicate the 95% CI. *n*≥11 in all cases. *P*-values were calculated by two-tailed *t*-tests with Welch's correction: ****P<0.0001, ***P<0.001.

Having observed activation of the unfolded protein response in CIN cells, we wished to test whether adding proteins that were difficult to fold would lead to aggregate formation and cell damage under these conditions (Eenjes et al., 2016). To observe protein aggregation in CIN cells, we expressed proteins containing polyglutamine (polyQ) repeats of different lengths (CAG55 and CAG91) that can form aggregates (van Eyk et al., 2012; Landrum and Wetzel, 2014). We observed greatly increased aggregation of myc-tagged CAG91 in CIN cells compared to the wild-type control (Fig. 2B), consistent with CIN cells having difficulty managing this additional protein folding burden. To test the effect of this burden on CIN cell survival, we measured Acridine Orange (AO) staining (Shaukat et al., 2012). Increased Acridine staining was observed in CIN cells expressing either CAG55 or CAG91 compared to their controls (Fig. 2C,D). Our data suggests that CIN cells suffer protein folding stress so that chaperones are incapable of resolving additional stress from aggregation-prone polyQ proteins.

### Overexpression of antioxidants rescues the effect of protein aggregation in CIN cells

ROS production has been associated with ER stress and the unfolded protein response (Cao and Kaufman, 2014). Oxidative stress causes the formation of oxidatively modified proteins that tend to aggregate in cytosol and trigger tumour progression (Kim et al., 2010). In order to test our hypothesis that the protein aggregation in CIN cells is ROS-dependent, we measured the effect of overexpression or depletion of the antioxidant enzyme catalase together with polyQ repeats. Catalase only directly removes hydrogen peroxide, not other reactive oxygen species, but we have previously noted that this can significantly affect CIN cell fates (Shaukat et al., 2015). We observed a significant decrease in polyQ protein aggregation in CIN wing discs of larvae co-expressing UAS-catalase and polyQ repeats compared to CIN discs expressing polyQ repeats only (Fig. 2E). We also observed an increase in aggregation when catalase was depleted by RNAi in CIN cells expressing polyQ protein. These results suggest that ROS contributes to protein folding stress in CIN cells which makes them vulnerable to the addition of hard-to-fold proteins.

### Effect of nucleotide interventions on CIN cells

**Fig. 3.**
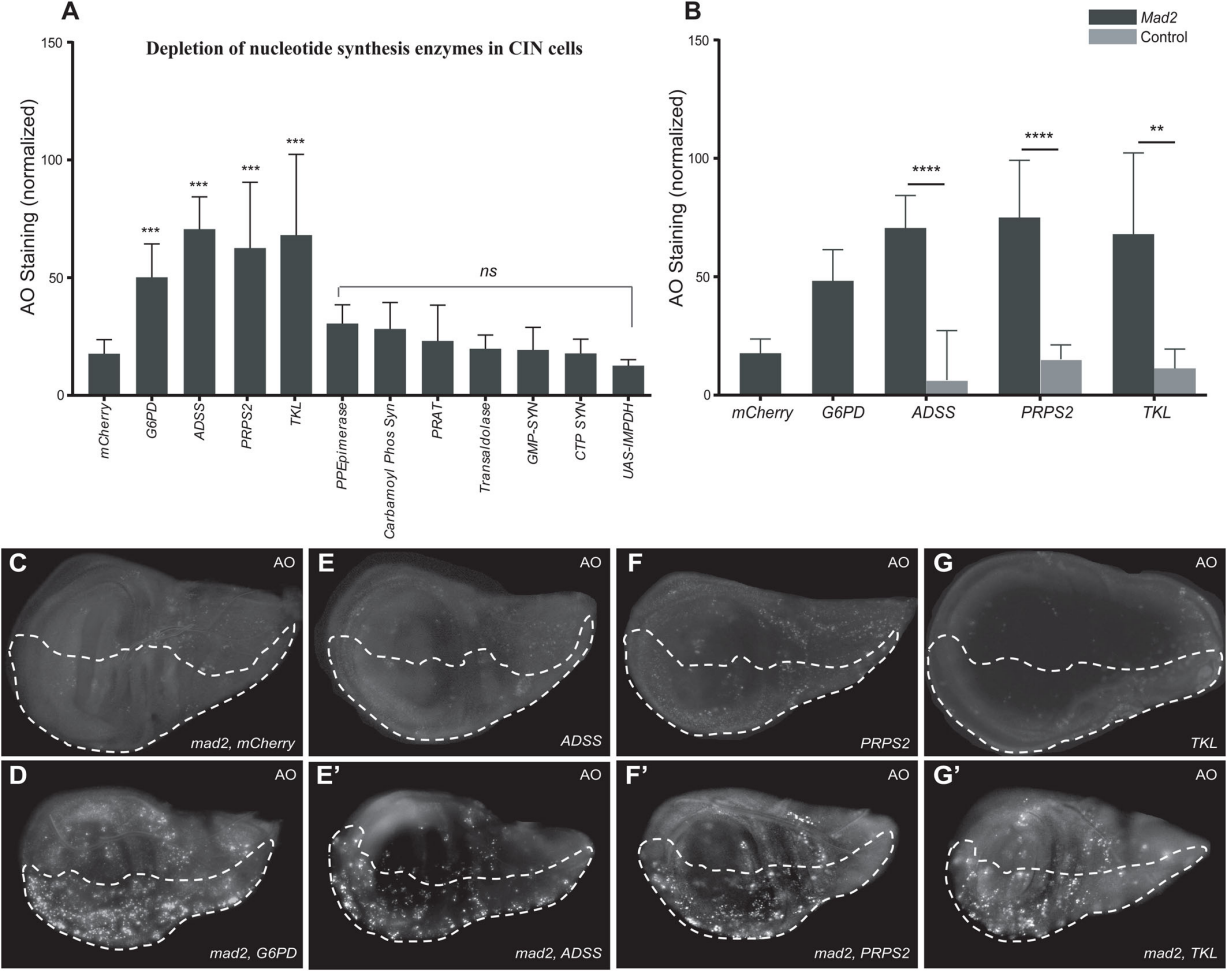
**Nucleotide synthesis gene knockdowns affect CIN cells.** (A) A quantitative analysis of Acridine Orange (AO) staining in third instar larvae wing discs knocked down for nucleotide synthesis enzymes. The y axis shows the normalized AO signals obtained by the subtracting the mean value of the control region from the affected region for each wing disc. Error bars represent 95% confidence intervals (CIs), *n*≥8 in all cases. *P*-values were calculated by two-tailed *t*-tests with Welch's correction: ***P<0.001. All tests compare candidate^RNAi^ *mad2*^RNAi^ with the negative control *mCherry*^RNAi^ *mad2*^RNAi^. (B) Quantification of AO staining in candidate^RNAi^ imaginal wing discs with and without CIN (see C. below). The y axis shows the normalized AO signals obtained by the subtracting the mean value of the control region for each wing disc. Knockdown of the candidate in non-CIN cells is represented by grey bars. Candidate knockdowns in CIN cells are represented by black bars. Error bars represent 95% CIs, *n*≥8 in all cases. *P*-values were calculated by two-tailed *t*-tests with Welch's correction: ****P<0.0001, **P<0.01. (C-G) AO staining of the third instar larvae wing discs. The dotted regions (*engrailed*>*Gal4*, *UAS-CD8-GFP*, with or without *UAS-mad2^RNAi^*) show the posterior compartment of the wing disc in which the candidates were knocked down, while the remainder of each wing disc is a wild-type internal control (C), negative control *mCherry mad2*^RNAi^ (D), positive control *G6PD mad2*^RNAi^ showing high levels of AO staining (E), *ADSS*^RNAi^ (E′), *ADSS*^RNAi^ *mad2*^RNAi^ (F), *PRPS2*^RNAi^ (F′), *PRPS2*^RNAi^ *mad2*^RNAi^ (G), *TKL*^RNAi^ (G′) and *TKL*^RNAi^ *mad*2^RNAi^. Knockdown of *ADSS*, *PRPS2* and *TKL* in CIN cells show high AO staining as compared to their knockdown in wild-type cells.

Like protein folding stress, nucleotide stress has been linked to CIN and cancer development (Bester et al., 2011). Nucleotide pool disequilibrium can result in genetic abnormalities and oncogenic transformation (Aird and Zhang, 2015). To identify the genes from nucleotide synthesis pathways that could be involved in regulating the fate of CIN cells, we tested a range of genes affecting purine biosynthesis (ADSS, PRPS2, GMP Synthetase, IMP dehydrogenase, PRAT), pyrimidine biosynthesis (CTP Synthetase, Carbamoyl Phosphate Synthetase) and the pentose phosphate (PP) pathway (TKL, Transaldolase, PP Epimerase). We initially measured their effect on CIN cells using RNAi to deplete the nucleotide pathway candidates in third instar larval wing discs with CIN induced by Mad2 depletion (Fig. 3A). We selected the candidates based on their AO phenotype, a cell viability assay that we have previously used to identify genes such as the positive control *G6PD*, that are required for CIN cell survival (Shaukat et al., 2012). The knockdown of ADSS, PRPS2 and TKL gave significantly elevated AO staining in the CIN region, while candidate depletion in non-CIN cells gave little or no AO, similar to the negative control, in this case RNAi to a gene not expressed in *Drosophila* (mCherry) (Fig. 3C-G). Quantification of AO signals from nucleotide pathway candidates with or without CIN showed elevated AO staining in CIN cells compared to non-CIN cells (Fig. 3B). These results suggest that CIN cells are sensitive to defects in nucleotide synthesis.

### Effect of depletion of nucleotide synthesis enzymes on oxidative stress

**Fig. 4.**
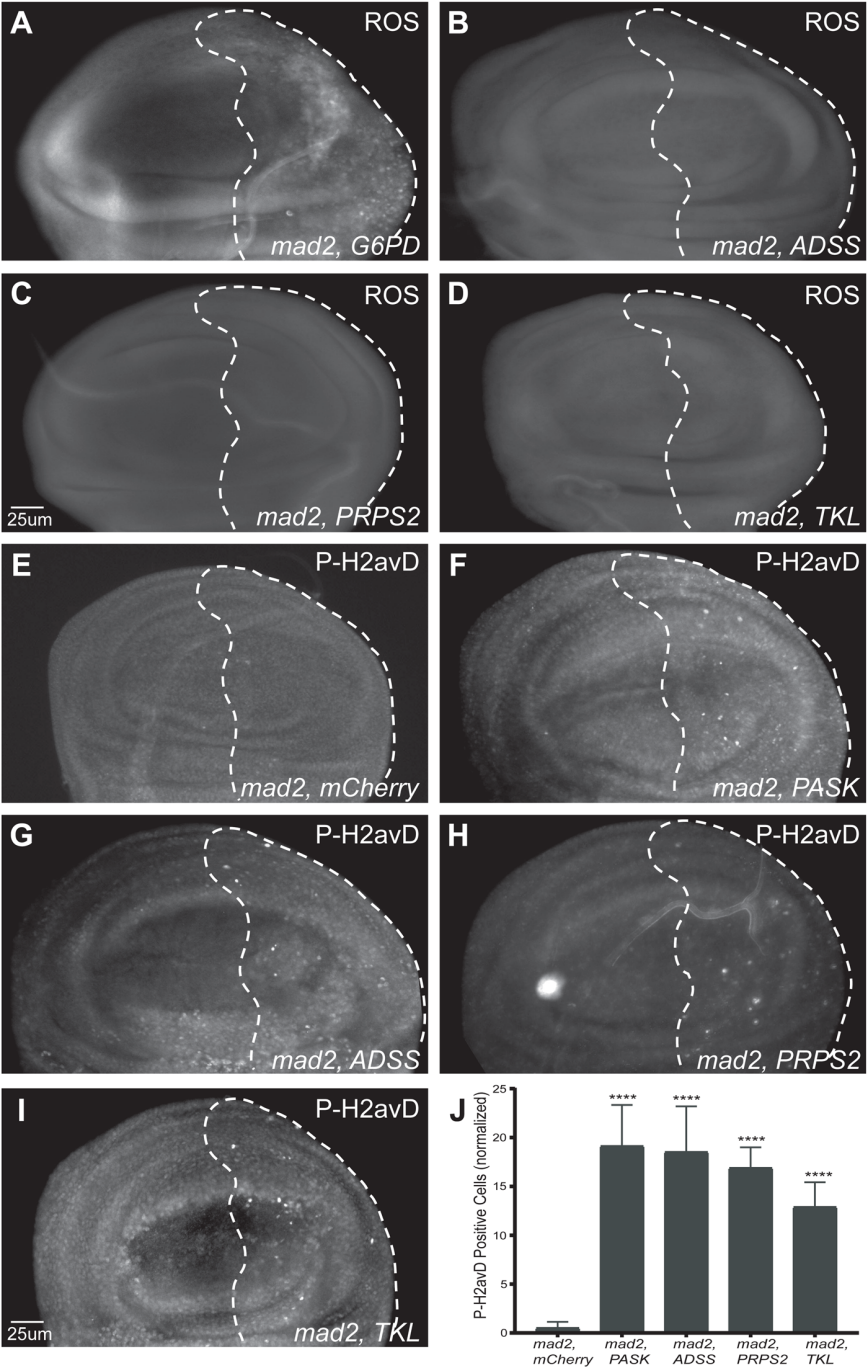
**The effect of depletion of nucleotide synthesis enzymes on oxidative stress and DNA damage in CIN cells.** Reactive oxygen species (ROS) levels were visualized by CellRox staining in third instar larval wing discs. Images show the anterior/posterior boundary of a representative wing disc pouch region from each genotype. The dotted line shows the *engrailed*-Gal4 expressing knockdown area in each image and left of the dotted line is wild type. (A) *G6PD*^RNAi^ *mad2*^RNAi^ positive control showing elevated ROS levels (B-D). We did not detect elevated ROS when nucleotide synthesis candidates were depleted in CIN cells. The experiment was repeated twice. (E-I) Third instar larvae wing discs were stained with anti-phosphorylated H2AvD (γH2AX) antibody staining to measure the level of DNA damage. The dashed line indicates the posterior compartment expressing (G) *ADSS*^RNAi^, (H) *PRPS2*^RNAi^, (I) *TKL*^RNAi^ in CIN cells, (E) negative control *mCherry^RNAi^* and (F) positive control *PASK*^RNAi^. The unmarked anterior compartment is wild type in each disc. (J) A quantitative analysis of P-H2AvD staining in third instar larval wing disc knockdowns of nucleotide synthesis enzymes in CIN cells. The y axis shows the number of P-H2AvD positive puncta cells in the engrailed-driven knockdown region, normalized by subtracting the number of stained cells in the control region for each disc. Elevated rates of P-H2AvD staining were seen when nucleotide synthesis enzymes were depleted in CIN cells. Error bars represent 95% CIs, *n*≥8 in all cases. *P*-values were calculated by two-tailed *t*-tests with Welch's correction, ****P<0.0001. All tests compare candidate^RNAi^ *mad2*^RNAi^ with *mCherry*^RNAi^ *mad2*^RNAi^.

We have previously found that disruptions to glucose metabolism in CIN cells not only increase Acridine staining, but also generate ROS and cause apoptosis (Shaukat et al., 2015). We carried out ROS assays to examine whether depletion of nucleotide synthesis enzymes may also elevate oxidative stress in CIN cells. We found that that knockdown of ADSS, PRPS2 and TKL gave no detectable increase ROS in CIN cells, in contrast to the positive control G6PD (Fig. 4A-D; Fig. S1C). These results suggest that depletion of nucleotide synthesis enzymes does not affect CIN cells by increasing ROS production.

### DNA damage in response to nucleotide synthesis enzyme depletion in CIN cells

Low nucleotide pools are known to cause replication stress and DNA damage in dividing cells (Kohnken et al., 2015). We next examined DNA damage by using anti-P-H2avD (γH2aX) antibody staining of larval wing discs with CIN in which nucleotide candidates had been knocked down (Fig. 4E-I). Quantification showed that depletion of ADSS, PRPS2 and TKL in CIN cells gave an elevated level of DNA damage, compared to the *mad2-*RNAi, *mCherry*-RNAi negative control (Fig. 4J). As expected, these data confirm that depletion of nucleotide synthesis enzymes caused DNA damage in CIN cells, consistent with decreased nucleotide availability.

### Depletion of nucleotide synthesis enzymes does not result in cell death in CIN cells

**Fig. 5.**
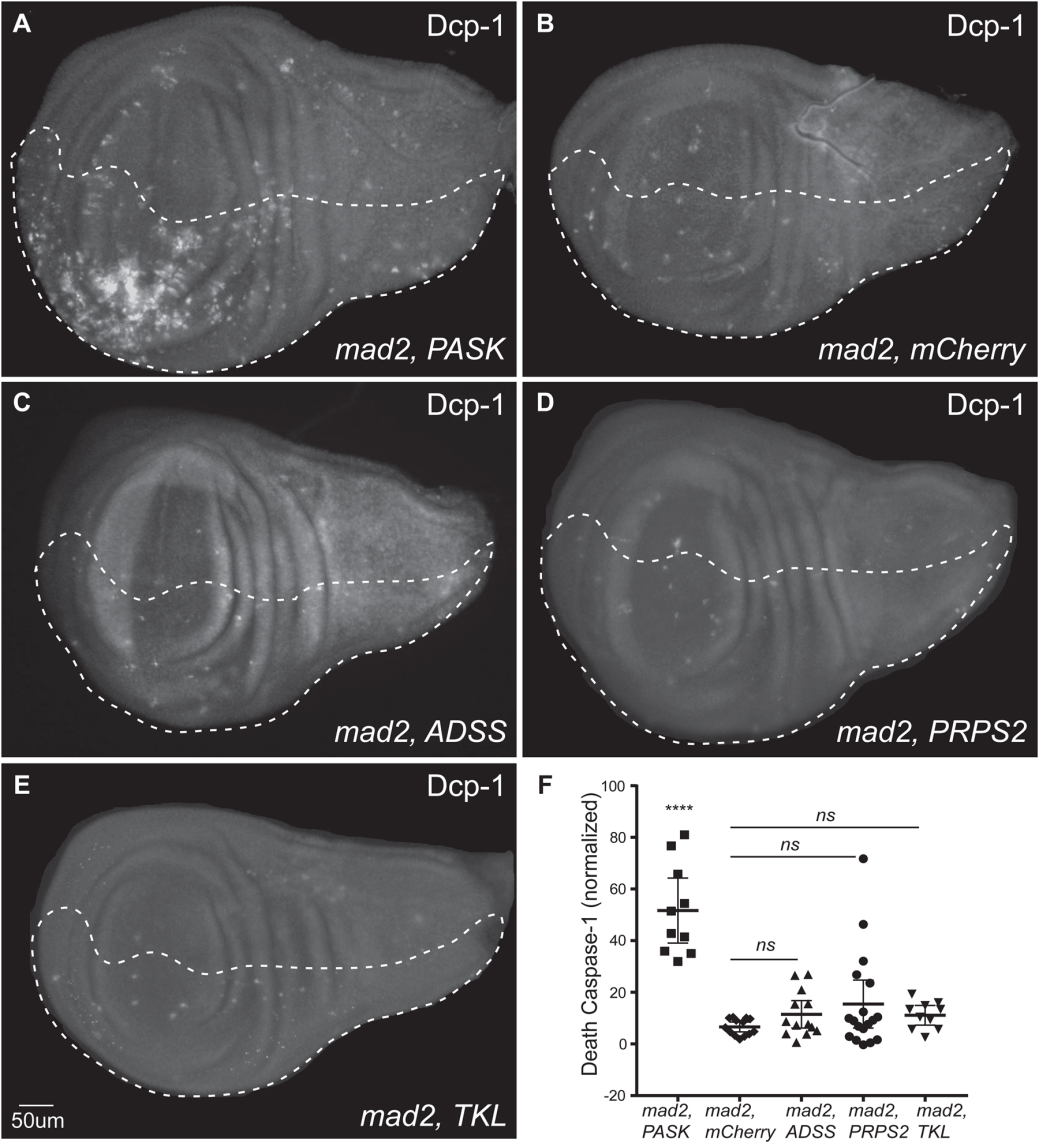
**Depletion of nucleotide synthesis enzymes did not cause apoptosis in CIN cells.** Death caspase-1 antibody was used to test for apoptosis in wing discs in which candidates were knocked down in CIN cells (dashed region). (A) Positive control *PASK mad2*^RNAi^ showed elevated apoptosis; (B) negative control *mCherry mad2*^RNAi^, (C) *ADSS*^RNAi^ *mad2*^RNAi^, (D) *PRPS2*^RNAi^ *mad2*^RNAi^ and (E) *TKL*^RNAi^ *mad2*^RNAi^ did not. (F) Quantification of Dcp-1 staining of positive control and candidate-RNAi imaginal wing discs. The y-axis shows the Dcp-1 stained cells in the engrailed driven region normalized by subtracting the number of stained cells in control region of each wing disc. The error bar indicates 95% CIs, *n*≥10 in all cases. The *P*-values were calculated by two-tailed *t*-tests with Welch's correction, ****P<0.0001. ns, non-significant. No significant elevation in apoptosis was seen when nucleotide synthesis enzymes were depleted in CIN cells. The experiment was repeated twice and confirmed by using anti-cleaved Caspase3 antibody staining (Fig. S1).

Having observed elevated Acridine staining and DNA damage, we expected that apoptosis might be elevated in CIN cells depleted for nucleotide synthesis enzymes (Abrams et al., 1993). To visualize apoptosis, we used anti-cleaved-Caspase 3 and anti-Dcp-1 antibody staining of wing discs. The levels of these antibody stainings were not elevated in double knockdowns of nucleotide candidates with Mad2 (Fig. 5B-E; Fig. S1) unlike the positive control (*mad2*-RNAi, *PASK-*RNAi) which generates ROS, DNA damage and apoptosis in CIN cells (Shaukat et al., 2015). Quantification of Dcp-1 signals from nucleotide candidates with CIN showed no significant increase in cell death compared to the *mad2*-RNAi, *mCherry-*RNAi negative control (Fig. 5F). However, cell death could have been caused by a caspase independent mechanism such as necrosis. We used Propidium Iodide (PI) staining to test for necrosis when our candidates were depleted in CIN cells. Consistent with the apoptosis data, these knockdowns in CIN cells were found to be negative for PI staining compared to the positive control (*JNK*^RNAi^, Fig. S2). Together, these results suggest that that depletion of nucleotide synthesis enzymes does not cause cell death either by apoptosis or necrosis in CIN cells. This surprising finding contrasts with the high levels of apoptosis induced by disrupting glucose metabolism in CIN cells (Shaukat et al., 2015).

### Effect of depletion of nucleotide synthesis enzymes on the cell cycle

We observed a significant increase in DNA damage when our nucleotide candidates were depleted in CIN cells, however we did not see cell death. Based on this finding, we hypothesized that this DNA damage could have led to cell cycle arrest in CIN cells. To test this hypothesis, we used Phospho-Histone 3 (PH3) antibody staining as a marker for scoring mitotic cells. We tested whether loss of our nucleotide candidates in CIN cells could prevent cell cycle progression by scoring the mitotic cells from larval wing discs of nucleotide candidates knocked down in cells with and without CIN. We did not observe any significant increase or decrease in PH3 staining in knockdowns of ADSS, PRPS2 and TKL in CIN or non-CIN cells (Fig. S3), suggesting that depletion of nucleotide synthesis enzymes does not arrest the cell cycle even in CIN cells showing DNA damage. Consistent with this, we did not see any change in S-phase duration as measured by EdU incorporation (Fig. S3C).

### Effect of depletion of nucleotide synthesis on activation of autophagy in CIN cells

**Fig. 6.**
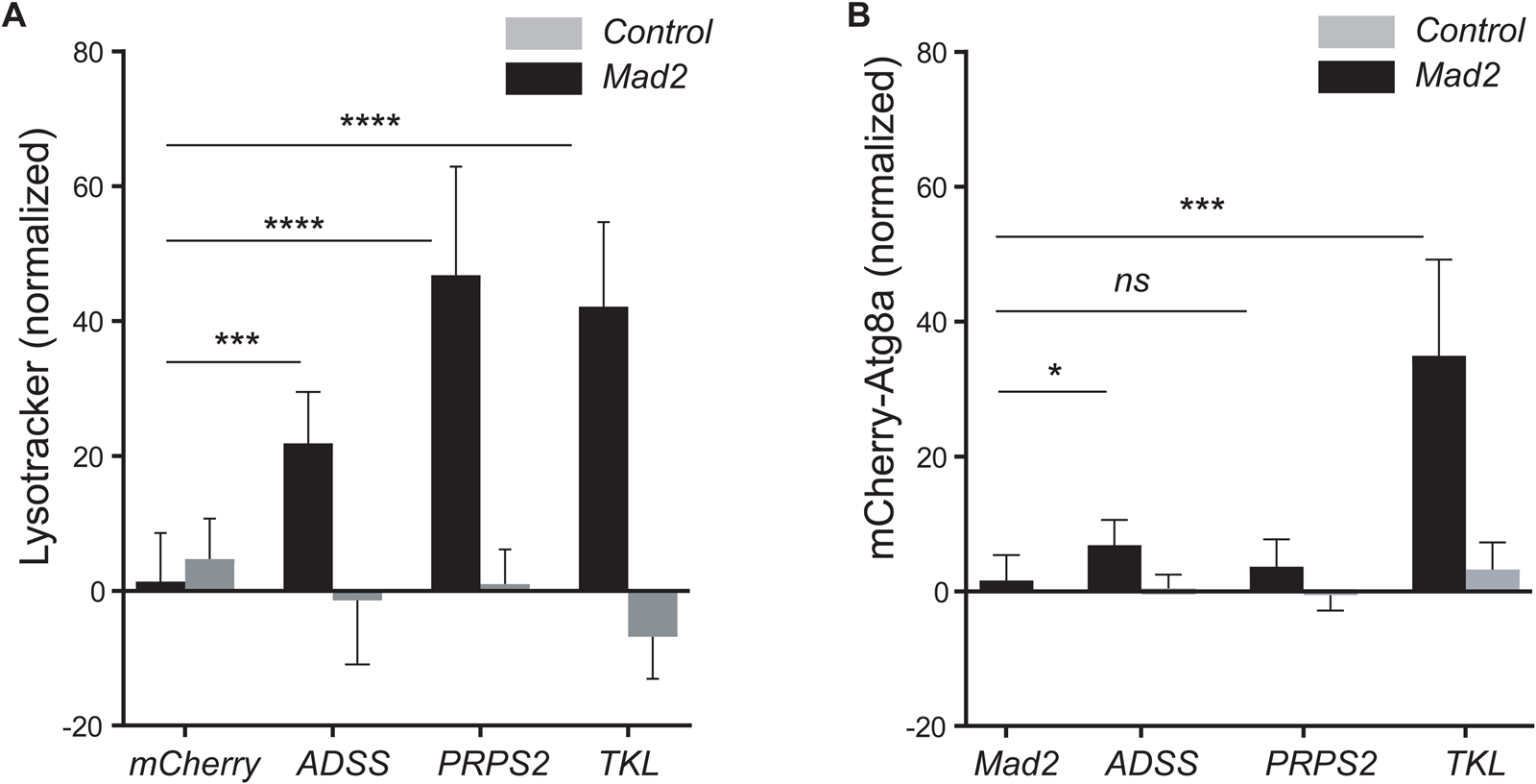
**Nucleotide candidate knockdowns caused the accumulation of lysosomes, but not always autophagy, in CIN cells.** Lysotracker staining was used to detect lysosomes. Quantification of Lysotracker staining is shown in (A). The y-axis shows the number of Lysotracker puncta in the *en*-driven knockdown region, normalized by the subtracting the number of puncta in the control region for each wing disc. The black bars represent the candidate knockdowns in CIN cells (*en*>*mad2*^RNAi^) and grey bars represent the candidate knockdowns in wild-type cells. Error bars indicate 95% CIs, *n*≥8 in all cases. The *P*-values were calculated by two-tailed *t*-test with Welch's correction: ****P<0.0001, ***P<0.001. Representative images are shown in Fig. S4. (B) Quantification of the level of mCherry-Atg8a puncta, measured as for Lysotracker. In all cases *n*≥10 and error bars show 95% CIs. The *P*-values were calculated using two-tailed *t*-tests with Welch's correction: ***P<0.001, *P<0.05; ns, non-significant. Representative images for this data are shown in Fig. S4.

It is known that autophagy is activated in CIN cells, and is needed for their survival (Liu et al., 2016). We wished to examine the effect of depleting nucleotide candidates on the activation of autophagy in CIN cells, to see if this could explain the Acridine staining, which normally stains the lysosomes in apoptotic cells (Abrams et al., 1993). We used Lysotracker staining and found that, like Acridine, it was elevated when any of the nucleotide candidates were depleted in CIN cells, relative to non-CIN controls (Fig. 6A; Fig. S4). We used a tagged form of Atg8a to confirm whether the high Lysotracker staining observed was due to activation of autophagy in knockdowns of ADSS, PRPS2 and TKL in CIN cells. Unlike the Lysotracker staining, only knockdown of TKL gave robust Atg8a puncta formation in CIN cells (Fig. 6B; Fig. S4). However, we did not observe Atg8a puncta in knockdown of ADSS or PRPS2 in CIN cells (Fig. 6B; Fig. S4). We expect that knockdowns of ADSS and PRPS2 decrease the rate of Adenosine Mono-Phosphate (AMP) synthesis and AMP is used to maintain phosphorylated AMPK to activate autophagy (Choi and Lee, 2011). However, increased Lysotracker staining in these candidates suggest that in absence of autophagy, lysosomes work hard to compensate.

### Feeding rATP and dATP rescue some AO phenotypes in CIN cells

**Fig. 7.**
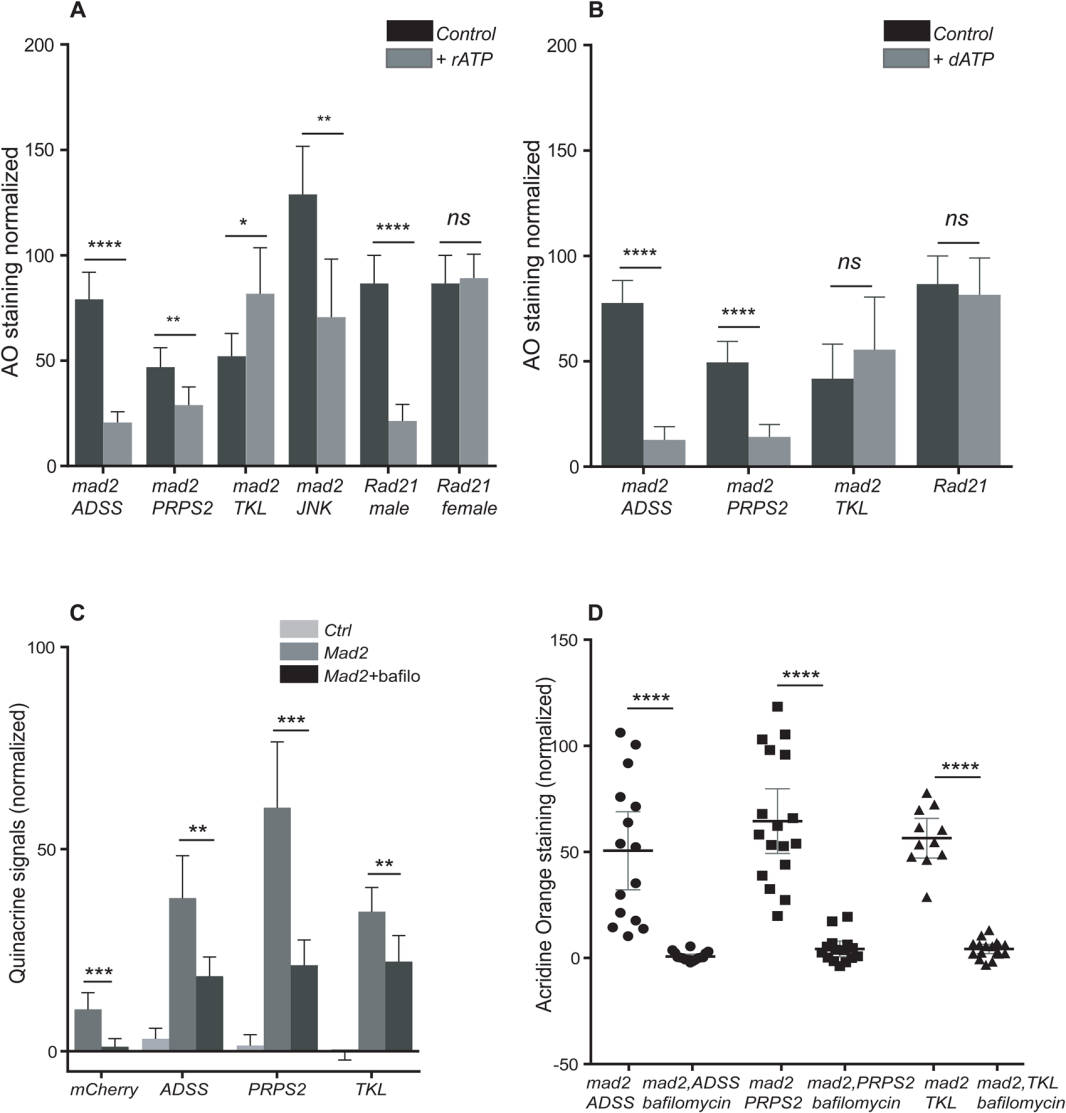
**Feeding ATP to larvae depleted for some nucleotide synthesis enzymes rescued their AO phenotype.** (A) Graph showing that depletion of *ADSS*, *PRPS2* or *JNK* in CIN cells (induced by *mad2*^RNAi^) showed high AO staining, which was rescued by feeding the larvae with rATP (1 mM). No rescue was observed in ATP fed larvae of genotype *TKL*^RNAi^ *mad2*^RNAi^. Male larvae with high CIN induced by Rad21 depletion (UAS-*rad21*^RNAi^, UAS-*Dicer2*) also showed high AO staining that could be rescued by rATP feeding, while feeding ATP to female larvae of the same genotype did not show reduction in their AO phenotype. Candidate knockdowns in CIN cells are represented by black bars and rATP fed candidate knockdowns in CIN cells are represented by grey bars. The error bars represent the 95% CIs, *n*≥10 in all cases. The experiment was repeated three times. Representative images are shown in Fig. S6. (B) Feeding the larvae with dATP (1 mM) rescued the AO phenotype in ADSS and PRPS2 knockdowns in CIN cells (grey bars) as compared to their controls (black bars). No rescue was observed in dATP fed larvae of genotypes *TKL*^RNAi^*mad2*^RNAi^ and in high-CIN larvae (UAS-*rad21*^RNAi^, UAS-*Dicer2*). The error bars represent the 95% CIs, *n*≥10 in all cases. The *P*-values were calculated by two-tailed *t*-tests with Welch's correction: ****P<0.0001, **P<0.01, *P<0.05; ns, non-significant. This experiment was repeated twice. Representative images are shown in Fig. S5. (C) Graph showing Quinacrine (QA, a marker for lysosomal ATP) staining on larval wing discs. High QA staining was observed, when nucleotide synthesis candidates were depleted in CIN cells (dark grey bar) as compared to candidates knocked down in non-CIN cells (light grey bar). Wing discs were incubated with bafilomycin (150 nM) for 30 min and then stained with QA (15 uM). Candidates' knockdown in CIN cells (black bar) show significantly lower QA staining after bafilomycin treatment. Quantifications show the normalized grey value of staining, which is obtained by subtracting the mean grey value of wild type from the affected region of each disc. Error bars indicate 95% CIs, *n*≥10 in all cases. The *P-*values were calculated by two-tailed *t*-test with Welch's correction: ****P<0.0001, ***P<0.001, **P<0.01; ns, non-significant. Representative images are shown in Fig. S6. (D) Graph showing that blocking v-ATPase by bafilomycin (75 nM) inhibits the AO phenotype in nucleotide candidate knockdowns in CIN cells. When wing discs of candidate knockdowns in CIN cells were incubated with bafilomycin for 30 min it significantly reduced the AO staining in these discs compared to controls. Quantifications show the normalized grey value of staining, obtained by subtracting the mean grey value of wild type from the mean of the affected region of each disc. The error bars represent 95% CIs, *n*≥12 in all cases. The *P*-values were calculated by two-tailed *t*-tests with Welch's correction. This experiment was repeated twice. Representative images are shown in Fig. S6.

Depletion of ADSS and PRPS2 should decrease the level of AMP synthesis, which will further lead to depletion of ATP. So, we examined whether feeding rATP or dATP to larvae would have an effect on the AO phenotype. First, we fed rATP to larvae knocked down for ADSS, PRPS2 and TKL in cells with CIN induced by Mad2 depletion. We observed that feeding rATP significantly rescued the AO phenotype when ADSS or PRPS2 were depleted in CIN cells. (Fig. 7A; Fig. S5). The same rescue of ADSS and PRPS2 was seen when larvae were fed dATP (Fig. 7B; Fig. S5). However, no rescue was seen when TKL depleted larvae were fed either nucleotide (Fig. 7; Fig. S5). This was consistent with the autophagy seen in the TKL knockdowns: these cells do not seem to be short of adenosine. We also tested a high CIN model (Rad21 depletion) to see if CIN might generate sensitivity to ATP levels in otherwise normal cells. Depletion of Rad21 leads to high levels of aneuploidy, AO, ROS and cell death (Liu et al., 2016). Surprisingly, we found that feeding Rad21 depleted larvae rATP strongly rescued the Acridine phenotype, but only in males (Fig. 7A; Fig. S5). dATP had no effect and females were unaffected by either nucleotide (Fig. 7B; Fig. S5). We conclude that elevated CIN can cause a loss of rATP, but do not know why females are less sensitive to this (c.f. Clemente-Ruiz et al., 2016). When AMP synthesis is compromised, our data suggest that CIN cells increase lysosome formation to compensate for the lack of autophagy.

### Lysosomal ATP levels in CIN cells

**Fig. 8.**
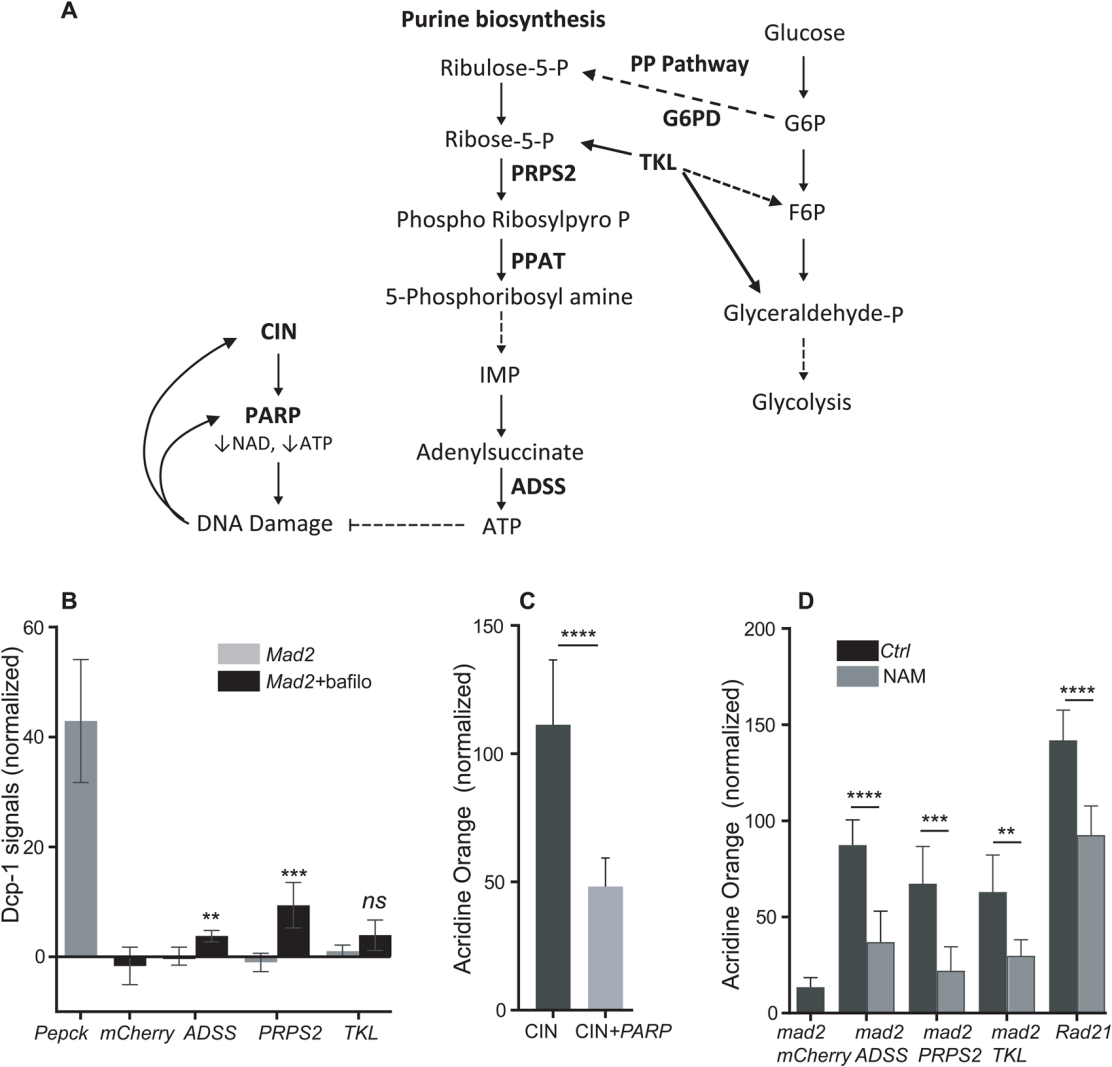
**Blocking v-ATPase causes cell death in nucleotide depleted CIN cells.** (A) A model for the effect of depletion of nucleotide synthesis enzymes on CIN cells. Loss of ADSS, PRPS2 or TKL decreases ATP synthesis and contributes to DNA damage in CIN cells, which are already ATP depleted by PARP activation. (B) Dcp-1 antibody staining was used to detect the level of apoptosis in bafilomycin treated wing discs of candidate knockdowns in CIN cells. The graph shows that depletion of ADSS and PRPS2 significantly increased the level of apoptosis in CIN cells after treatment with bafilomycin. Depletion of TKL caused a non-significant increase in apoptosis in bafilomycin treated CIN cells. In all cases *n*≥10 and error bars show 95% CIs around the mean. The *P*-values were calculated by two-tailed *t*-tests with Welch's correction. Representative images are shown in Fig. S7. (C) Graph showing that PARP knockdown in CIN cells (induced by Rad21 depletion) significantly rescued the AO phenotype in these cells, which in this case we know represents apoptosis (Liu et al., 2015). Quantifications show the normalized grey value of staining obtained by subtracting the mean grey value of wild type from the affected half of each disc. In all cases *n*≥23 and error bars show 95% CIs around the mean. The *P*-values were calculated by two-tailed *t*-tests with Welch's correction. (D) Nicotinamide feeding rescued the AO phenotype caused by nucleotide synthesis enzyme depletion in CIN cells. Quantification showing that the AO phenotype of ADSS, PRPS2 and TKL depletion in CIN cells was significantly rescued by feeding the larvae with Nicotinamide (1 mM). A similar reduction was observed in high CIN cells (induced by Rad21 depletion). In all cases *n*≥12 the error bar show 95% CIs around the mean. The *P*-values were calculated by two-tailed *t*-tests with Welch's correction, ****P<0.0001, ***P<0.001, **P<0.01; ns, non-significant. This experiment was repeated twice. Representative images are shown in Fig. S8.

Effective lysosomal function is known to play a vital role in cancer progression and metastasis (Appelqvist et al., 2013). To examine the induction of lysosomes as a result of depletion of nucleotide synthesis enzymes in CIN cells, we used Quinacrine (QA), a marker for lysosomal ATP (Cao et al., 2014). Robust QA staining was observed in knockdowns of ADSS, PRPS2 and TKL in CIN cells relative to the inert *mCherry*-RNAi control (Fig. 8A; Fig. S6). This suggests that there is no lack of ATP inside lysosomes, although our feeding experiments argue that these cells lack ATP overall. In fact, we observed more QA staining in CIN cells than non-CIN controls (Fig. S6). A multi-subunit lysosomal membrane protein, v-ATPase, maintains lysosomal acidification by importing protons from the cytoplasm to the lysosomal lumen (Collaco et al., 2013), and is required for ATP transport into the lysosome (Mauvezin et al., 2015). Blocking v-ATPase with bafilomycin significantly reduced the QA signal (Fig. 8A) as expected. By blocking lysosome acidification, this inhibition of v-ATPase also strongly rescued the AO phenotype seen in CIN cells depleted for nucleotide synthesis enzymes (Fig. 8B; Fig. S6).

### Lysosomes are needed for CIN cell survival

It has been reported that advanced cancers exploit autophagy-lysosomal pathways to avoid cell death. Recent studies indicate that compromised lysosomal function in mammalian cell lines resulted in cell death (Cao et al., 2014). Moreover, having previously found that autophagy is needed for CIN cell survival (Liu et al., 2016), we wished to investigate the role of lysosomes in determining the fate of CIN cells, particularly in the absence of autophagy. We treated CIN wing discs knocked down for our candidates with bafilomycin to block v-ATPase, and then stained the wing discs with Dcp-1 to measure the level of cell death. The positive control was depletion of PEPCK, which gives elevated apoptosis in CIN cells and tumours (Hussain et al., 2017). We found that bafilomycin treatment caused some cell death in CIN cells knocked down for ADSS or PRPS2 and to some extent TKL, but not in CIN cells alone (Fig. 8C; Fig. S7). These results suggest that in CIN cells, in the absence of autophagy, lysosomes were able to compensate but, if we also blocked lysosomes, the CIN cells died.

### Replacing NAD+ rescues the AO phenotype

We have shown that that depletion of ADSS and PRPS2 caused DNA damage in CIN cells and both rATP and dATP significantly rescued the AO phenotype. This led us to hypothesize that knockdowns of ADSS and PRPS2 depleted the level of adenosine, causing DNA damage during replication, which we would expect to then activate PARP as part of the repair process (Weaver and Yang, 2013). In normal cells, the depletion of nucleotide synthesis had no effect, so we wondered whether CIN cells were particularly vulnerable to PARP activation, which can further decrease ATP levels (Weaver and Yang, 2013). We found that cells with high CIN levels activated PARP to some degree even without nucleotide depletion (Fig. S8G). In these high CIN cells, we know that AO marks cell death (Liu et al., 2015), and we found that blocking PARP activation rescued a significant amount of that cell death (Fig. 8D). Because PARP consumes NAD+ and leads to ATP depletion (Zong et al., 2004), we predicted that providing extra NAD+ to high CIN cells might rescue the AO phenotype in these cells. To test our model, we fed larvae with nicotinamide (NAM), a precursor in the synthesis of NAD+ (Kirkland, 2012). We observed a significant reduction in the AO phenotype in wing discs when NAM was fed to larvae with decreased nucleotide synthesis (ADSS, PRPS2 or TKL) or with high CIN levels (Rad21) relative to controls (Fig. 8E; Fig. S8). We conclude that CIN cells tend to activate PARP, which decreases their levels of NAD+ and ATP, making them particularly vulnerable to interventions that further limit ATP availability. At moderate CIN levels, depleted adenosine does not kill the cell, but does limit autophagy, leading to an increased dependence on lysosomes, which then become critical for CIN cell survival.

## DISCUSSION

Aneuploidy is a state in which cells carry unbalanced genomes. Errors in chromosomal segregation lead to whole chromosome aneuploidy while segmental aneuploidy mainly originates due to defects and delays in the DNA replication and repair mechanism (Gordon et al., 2012; Janssen and Medema, 2013). Aneuploidy is a hallmark of cancer and has been shown to be poorly tolerated at the cellular and organismal level (Weaver and Cleveland, 2006). Aneuploidy can have a range of effects including aberrant cell growth, proliferation, proteotoxic stress and oxidative stress (Pfau and Amon, 2012). We have found that the induction of aneuploidy makes cells highly glycolytic and vulnerable to oxidative stress and they show DNA damage and apoptosis in response to metabolic interventions that do not damage normal cells (Shaukat et al., 2015). The mechanism that regulates the change in metabolism in response to aneuploidy is unclear, therefore, we wished to identify the stereotypical metabolic signature of aneuploidy. Aneuploidy is known to cause ER stress and oxidative stress in most organisms such as yeast, plants, mouse and human (Pfau and Amon, 2012; Sheltzer et al., 2012). Aneuploidy related impaired proliferation, altered protein folding and ER stress is thought to be due to the changes in protein stoichiometry from the aberrant chromosomes. In this study, increases in chaperone levels (Hsc70, Hsp83) and ER stress markers (XBP1) were observed in aneuploid cells. We also observed that ER stress can cause ROS by calcium release in aneuploid cells. Our data confirm that aneuploid cells suffer protein folding stress so that chaperones are incapable of resolving additional stress (i.e. aggregation prone polyQ proteins). Successful aneuploidy-tolerant cells must be able to enhance repair and reduce oxidative stress and cell death, so we wished to identify the regulation of pathways required for aneuploidy tolerance. Autophagy is known to be induced by ER stress and ROS, and is a plausible mechanism for aneuploidy tolerance (Sheltzer, 2013). Aneuploid cells have three related defects that autophagy could help to moderate: proteotoxic stress, defective mitochondria and oxidative damage (Li et al., 2017; Shaukat et al., 2015). We have shown that aneuploid cells are dependent on autophagy of defective mitochondria to tolerate their aneuploidy (Liu et al., 2016). We further analysed the production of ROS and ER stress in aneuploid cells. Previous studies revealed that in ER-stressed cells, Ca^2+^ released from the ER is taken up by mitochondria and releases cytochrome c, which inhibits complex III of the electron transport chain and increases ROS production. Moreover, increased Ca^2+^ in the mitochondria stimulates Krebs cycle dehydrogenases, thus increasing the oxygen consumption and ROS production. Mitochondrial Ca^2+^ also activates nitric oxide synthase, whose product disturbs the ETC and enhances ROS generation (Brand, 2010; St-Pierre et al., 2002). However it is not clear whether the ER stress generates the ROS or ROS generates the stressed ER or both. In this study, we addressed this question and showed that ROS contributes to protein folding defects in aneuploid cells, which could be significantly rescued by adding antioxidants.

We have previously observed that aneuploid cells have overactive mitochondria, which produce ROS and hence oxidative damage to proteins, lipids and DNA and damage to macromolecules (Shaukat et al., 2015), which we now find causing protein folding defects and ER stress. Moreover, effective repair mechanisms, autophagy and antioxidant levels are required to tolerate the deleterious effects of aneuploidy.

Further, we tested the effect of nucleotide stress on CIN cells. We carried out preliminary screenings based on AO staining and tested the range of genes affecting purine biosynthesis, pyrimidine biosynthesis and the PP pathway. We found that the knockdown of ADSS, PRPS2 and TKL gave significantly higher AO staining in a CIN background. These nucleotide candidates play a central metabolic role in the synthesis of nucleotides. The PRPS enzyme adds a pyrophosphate group from ATP to ribose-5-phosphate generated from the PP pathway to produce a nucleotide precursor called 5-phosphoribosyl-1-pyrophosphate (PRPP). PRPP is a substrate for all nucleotide salvage pathway enzymes, as well as for the rate-limiting enzymes of purine and pyridine biosynthesis. Modulation of PRPS levels by either knockdown or overexpression respectively inhibit or potentiate nucleotide production, suggesting that levels of PRPP may be sufficient to govern the overall rate of purine metabolism (Cunningham et al., 2014). ADSS is downstream of the *de novo* pathway and it is a vital component of the *de novo* pathway as well as the salvage pathway. Similarly, ADSS is a highly conserved enzyme among all living organisms, it converts inosine monophosphate (IMP) to AMP as part of ATP biosynthesis.

The high AO phenotypes in nucleotide candidate knockdowns in CIN cells suggest that CIN cells are sensitive to changes in nucleotide levels. In addition, increased Lysotracker and DNA damage phenotypes were observed when nucleotide synthesis enzymes ADSS, PRPS2 and TKL were depleted in a CIN background. However, we did not see any involvement of ROS or cell cycle arrest in this case. Cell death either by apoptosis or necrosis was also not observed when these candidates were depleted in CIN cells. Therefore, we expected that the autophagy pathway was being activated. Autophagy is known to be activated in response to various stresses including nutrient starvation (Jiang and Mizushima, 2014; White, 2015). We have shown that CIN cells are dependent on the autophagy of mitochondria to tolerate their aneuploidy and avoid triggering innate immune signalling (Liu et al., 2016). For example, blocking the autophagy pathway in CIN cells led to an increased number of dysfunctional mitochondria, increased levels of oxidative stress, DNA damage and apoptosis, while enhancing autophagy could reduce the level of ROS and apoptosis. So we hypothesized that autophagy might be triggered in response to nucleotide depletion. Surprisingly, autophagy was not detected in ADSS and PRPS2 deficient CIN cells. However, elevated puncta of tagged Atg8a were observed in TKL deficient CIN cells, suggesting that autophagy is activated in TKL deficient CIN cells. TKL is a pivotal enzyme of the non-oxidative arm of the PP pathway. It catalyses the common two-substrate reactions in order to generate glyceraldehyde-3-phosphate and fructose-6-phosphate, which re-enter glycolysis. When cells are in need of nucleotides, the PP pathway produces ribose via the oxidative as well as the non-oxidative arm from fructose-6-phosphate and glyceraldehyde-3-phosphate. TKL1 also enhances the PP pathway flux for biosynthetic reactions, as the PP pathway generates ribose-5-phosphate for nucleic acid synthesis and NADPH for fatty acid synthesis and maintaining redox homeostasis to protect cells against oxidative stress and apoptosis (Diaz-Moralli et al., 2016; Vaughn and Deshmukh, 2008). Moreover, inhibition of TKL will suppress the PP pathway and interrupt the synthesis of important coenzymes such as ATP, CoA, NAD(P)+, FAD, and genetic material, RNA and DNA. We found that depletion of TKL in CIN cells triggered autophagy, which we interpret as a CIN cell survival response, based on previous work by ourselves and others (Santaguida et al., 2015; Liu et al., 2016).

Increased lysosome staining in ADSS and PRPS2 deficient CIN cells suggests lysosomal involvement in CIN cells. Lysosomes play a crucial role in cancer progression by regulating complex processes involving protein secretion, endocytic receptor recycling, energy metabolism and cell signalling (Appelqvist et al., 2013; Stingele and Storchova, 2013). Moreover, the autophagy–lysosome pathway is closely linked with the hallmarks of cancer including escaping cell death pathways, evading immune surveillance and deregulating metabolism (Hanahan and Weinberg, 2011). Advanced cancer cells are extremely reliant on effective lysosomal function. As a consequence, cancer progression and metastasis are related with unusual changes in lysosomal compartments, such as lysosome volume, composition, cellular distribution, and lysosomal enzyme activity, as compared with normal cells (Gocheva et al., 2006; Nishimura et al., 1998). Furthermore, when we blocked lysosomes, ADSS or PRPS2 depleted CIN cells showed increased cell death. The modest effect of bafilomycin on Tkl depleted CIN cells suggests that autophagy is not entirely able to clear the problems when lysosomes are blocked. These results suggest that in the absence of autophagy, lysosomes worked hard in these cells to compensate for the lack of autophagy and when we blocked lysosomes the CIN cells died. So, our model is that CIN cells use lysosomes to compensate for loss of autophagy when AMP is depleted.

Depletion of ADSS and PRPS2 decrease the synthesis of AMP which further leads to depletion of ATP, and we observed that in larvae knocked down for ADSS or PRPS2, feeding rATP and dATP significantly rescued the AO phenotype in CIN cells relative to their controls. These results suggest that CIN cells are sensitive to ATP changes. To further understand why ATP might be limited in CIN cells, we tested for PARP and found that it is upregulated in CIN cells. We also observed rescue of oxidative stress and cell death when we knocked down PARP in CIN cells. The PARP enzyme PARylates a variety of protein substrates and alters their interaction with DNA and other proteins. PARP is currently in clinical trials for DNA damage sensitive cancers because of its role in the DNA damage response (Weaver and Yang, 2013). PARP activation causes depletion of its substrate NAD+, which further leads to loss of ATP (Zong et al., 2004). We found that adding ATP or nicotinamide (a precursor in the synthesis of NAD+) rescued the AO phenotype in CIN cells. These results suggest that ATP and NAD+ are consumed for PARP activation and CIN cell survival.

Our model is that knockdowns of ADSS and PRPS2 in CIN cells deplete the nucleotide level which result in DNA damage. DNA damage activates PARP that causes a reduction in both the NAD^+^ and the ATP pool. CIN cells already lack ATP due to activation of PARP, and less ATP in CIN cells is paralleled by increasing AMP levels. High AMP levels, sensed by AMPK, should lead to activation of autophagy, which is required for CIN cell adaptation in a nutrient starved condition. However, loss of AMP by ADSS knockdown in CIN cells seems to prevent activation of autophagy. An elevated lysosomal phenotype in ADSS deficient CIN cells shows that in the absence of autophagy, lysosomes work hard to compensate for the autophagy defect, and this is required for the cells to survive. Overall, our results suggest that cellular homeostasis is significantly disrupted by aneuploidy, with defects originating from elevated ROS levels that exacerbate protein folding stress and damage DNA. Because DNA damage activates PARP, aneuploid cells are sensitive to levels of NAD+ and ATP, making this metabolic pathway a promising therapeutic option for the treatment of aneuploid tumours.

## MATERIALS AND METHODS

### *Drosophila* stocks

The fly stocks used in this study were obtained from Bloomington Drosophila Stock Center unless otherwise stated and are as follows:

*mad2*-RNAi [Vienna Drosophila Resource Center (VDRC) #47918], *Rad21*-RNAi (#36786), *ADSS*-RNAi (#33993), *PRPS2*-RNAi (#35619), *TKL*-RNAi (#32884), Ribulose-*Phosphate3-Epimerase*-RNAi (#42816), *Carbamoyl Phosphate Synthetase*-RNAi (#38332), *PRAT*-RNAi (#51492), *Transaldolase*-RNAi (#51709), *GMP Synthetase*-RNAi (#31055), *CTP Synthetase*-RNAi (#31752), UAS-*IMP dehydrogenase* (#11284), *JNK*-RNAi (VDRC #34138), *mCherry-Atg8a* (#37750), *PEPCK*-RNAi (#17725), *PASK*-RNAi (#3105), *G6PD*-RNAi (#12529), UAS-XBP1-GFP (#39719), Hsp83-GFP [Drosophila Genomics Resource Center (DGRC) #109-697], UAS-CAG55 and UAS-CAG91 (a gift from Prof. R. Richards, University of Adelaide), UAS-Catalase (#24621), PARP-RNAi (#35792), Parg-RNAi (#61333), UAS-GCaMP (#32236). The driver stock *engrailed* (*en*-*Gal4*) for gene expression in the posterior region of wing discs is Bloomington #30564.

### AO staining

Third instar larvae were dissected in phosphate-buffered saline (PBS), then discs were incubated in 1 mM AO for 2 min then transferred to a slide after a brief wash. The treated imaginal discs were immediately mounted under a bridged cover slip and imaged in PBS. For quantification, the AO stain was normalized by subtracting the wild-type region value from the test region value (*engrailed*-driven region). The background noise of all images was subtracted in ImageJ using a rolling ball radius of 10 pixels.

### PI staining

PI staining was used to measure caspase-independent cell death. Third instar larvae were dissected in PBS for imaginal discs, the collected imaginal discs were incubated in 3 uM PI for 5 min then transferred to a slide after a brief wash. Mounting, imaging and quantification was done similar to AO staining as described above.

### Lysotracker and Hoechst staining

Lysotracker staining was used to detect lysosomes in larval wing imaginal discs. The dissected imaginal discs were transferred from PBS and incubated in 1 uM Lysotracker (Lysotracker Red DND-99, Invitrogen) and 6 ug/ml Hoechst (Hoechst 33342, Sigma-Aldrich) for 5 min and then mounted to a slide with PBS for microscopy after a quick wash in PBS.

### QA staining

To identify lysosomal ATP level, we used QA staining on the wing discs of third instar larvae. Candidates were knocked down using *en-gal4* driver, approximately equal aged larvae were selected and wing discs were dissected out in PBS. Dissected discs were then incubated in 50 uM QA for 2 min. Mounting and imaging was done as for the AO staining described above.

### Oxidative stress assay

The level of ROS in CIN cells was measured using the fluorogenic probe CellRox Deep Red from Life Technologies according to manufacturer's recommendations. The third instar larvae were dissected in D22 media with pH 6.8. Then the dissected imaginal wing discs were transferred into 5 μM CellRox (in D22 media) for 20 min. The wing discs were quickly washed in PBS and fixed in 3.7% formaldehyde for 5 min then mounted in 80% glycerol for imaging.

### Calcium imaging

Intracellular calcium was detected by imaging of GCaMP3. Third instar wing discs were dissected in PBS, briefly fixed in 4% formaldehyde and mounted in 80% glycerol for imaging.

### Immunostaining

Immunostaining was used on dissected wing imaginal discs for different purposes. Third instar larvae were dissected in PBS for imaginal discs, then the collected imaginal discs were fixed in 3.7% formaldehyde for 20 min and then washed for 30 min in 0.2% PBST (1×PBS+0.2% Tween). The fixed imaginal wing discs were then blocked in PBSTF (1×PBS+0.2% Tween+5% fetal calf serum) for 30 min and stained with the primary antibody for overnight at 4°C. After staining with the primary antibody, the wing discs were washed in 0.2% PBST for 30 min then transferred to a secondary antibody solution for 2.5 h at room temperature in the dark. After 30 min washing in PBST, the wing discs were mounted in 80% glycerol for imaging. To detect parylation, discs mutant for the de-parylating enzyme *PARG* were used to improve the sensitivity of detection (Fig. S8).

The source and concentration of antibodies used are as follows:

Rabbit anti-cleaved caspase 3 (D175, 1:100; Cell Signalling #9661S), Rabbit anti-cleaved *Drosophila* Dcp1 (Asp216, 1:100; Cell Signalling #9578), Mouse anti-P-Histone3 (Ser10, 1:500; Cell Signalling #9706S), Rabbit anti-P-H2avD (1:500; Rockland, lot # 30352), Mouse anti-pADPr (PAR, Clone 10H, 1:100; Tulip Biolabs #1020/N).

The secondary antibodies used were goat anti-rabbit CY3 (1:100; Life Technologies), rhodamine anti-mouse (1:200) and rhodamine anti-rabbit (1:200, both Jackson ImmunoResearch).

### Drug treatments

For wing disc culture experiments, drugs were mixed with fly extract media, fly extract media was prepared by using Schneider's media (Invitrogen) 94%, whole fly extract 5% (Currie et al., 1988), Bovine Insulin (Sigma-Aldrich) (1 mg/ml) 0.5%, penicillin/streptomycin 1000 U/ml 0.5%.

For drug feeding experiments, drugs were mixed in standard fly food (semolina, yeast, molasses, agar, glucose, water, Tegosept and acid-mix) and were given to larvae when solidified. Drugs used in this paper are as follows:

6-Aminonicotinamide (6-ANA) 500 uM, Bafilomycin 150 nM, Adenosine 5′-triphosphate disodium salt hydrate (rATP) 1 mM, dATP PCR Grade, sodium salt 1 mM, Nicotinamide (NAM) 1 mM. All drugs were obtained from Sigma-Aldrich.

### Data analysis

Statistical analysis was done in Prism (GraphPad) using Dunnett's multiple comparisons and two-tailed *t*-test with Welch's correction. In all graphs, error bars show 95% confidence intervals. All microscopy was done on a Zeiss Axioplan2 microscope. Axiovision software (Carl Zeiss), Adobe Photoshop, Adobe Illustrator and ImageJ (http://fiji.sc/) were used for image processing and quantification.

## Supplementary Material

Supplementary information
