# Supplementary material for: Chromosomal instability causes sensitivity to protein folding stress and ATP depletion
Source: Biol Open. 2018 Oct 15;7(10):bio038000. doi: 10.1242/bio.038000 (PMC6215417; doi:10.1242/bio.038000)
Supplement: Supplementary information [file biolopen-7-038000-s1.pdf]

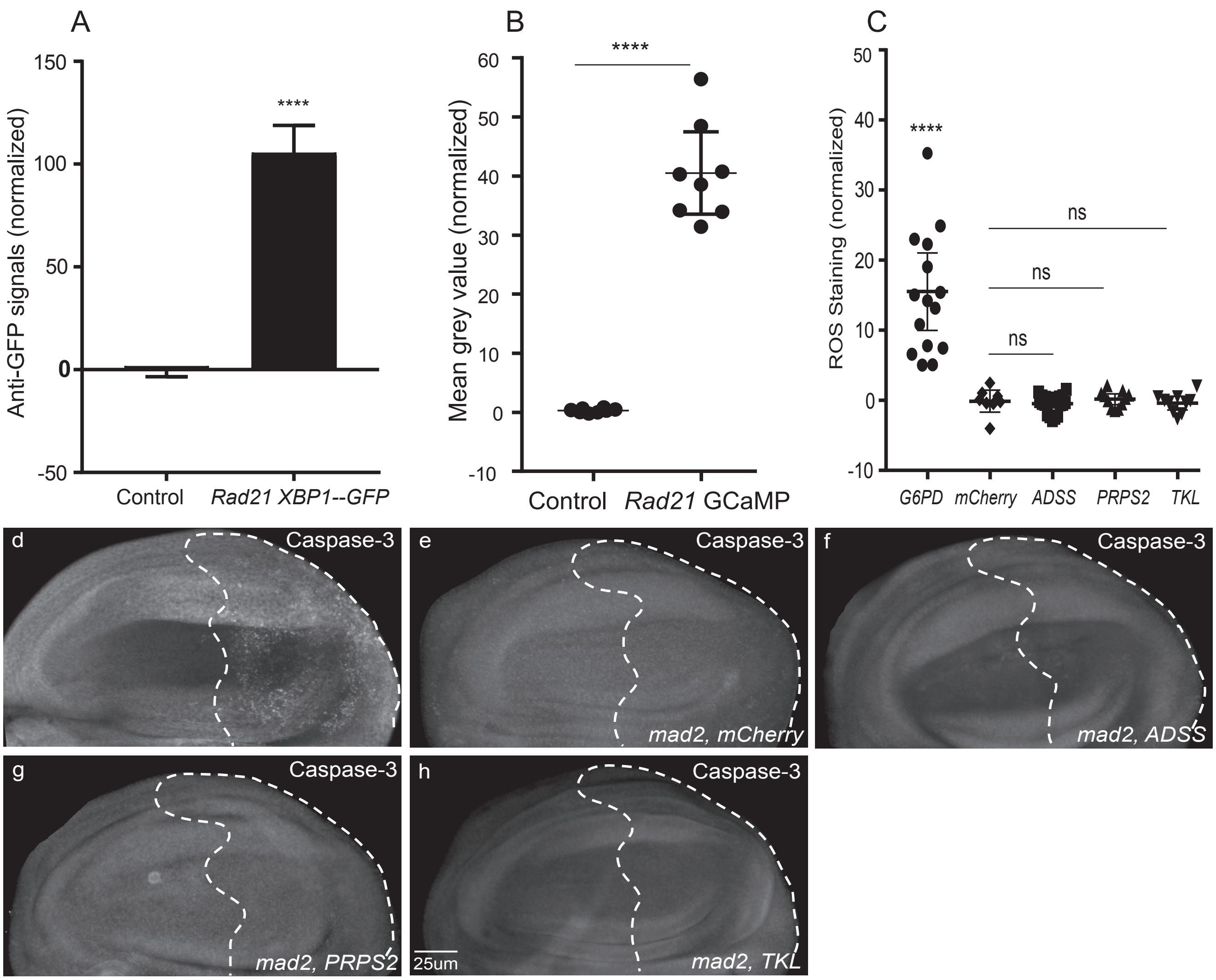

Figure S1: (**A**) Quantification of XBP1-GFP expression. Increased GFP levels which indicate XBP1-GFP were observed when CIN was induced by depletion of *en*>Rad21 (UAS-Rad21^{RNAi}, UAS-Dicer2) compared to the control *en*>UAS-XBP1-GFP. The y axis shows the normalized anti-GFP staining obtained by the subtracting the mean value of the control region from the mean of the affected region for each wing disc. The error bars indicate the 95% confidence interval, n≥9 in all cases. The p values were calculated by two-tailed t-tests with Welch's correction. (**B**) Quantification of GCaMP3 signals. Increased GCaMP signal was observed in *en*>Rad21 (UAS-Rad21^{RNAi}, UAS-Dicer2 relative to wild type controls. The y axis shows the normalized GCaMP3 signals obtained by the subtracting the mean value of the control region from the mean of the affected region for each wing disc. The error bars indicate the 95% confidence interval, n=8 in all cases. (**C**) Quantification of ROS staining. The y axis shows the normalized CellROX staining obtained by the subtracting the mean value of the control region from the mean of the affected region for each wing disc. The error bars indicate the 95% confidence interval, n≥8 in all cases. Genotypes tested were (*engrailed* driven) positive control *G6PD mad2*^{RNAi}, negative control *mCherry mad2*^{RNAi,} *ADSS*^{RNAi} *mad2*^{RNAi}, *PRPS2*^{RNAi} *mad2*^{RNAi}, *TKL*^{RNAi} *mad2*^{RNAi} (**d, e, f, g, h**) Caspase3-driven apoptosis was not detected when candidates were depleted in CIN cells. Anti-cleaved caspase 3 antibody was used to measure the apoptosis in third instar larval wing discs. In every disc, the unmarked region does not express RNAi constructs, while the dashed line shows the area affected by CIN (*mad2-RNAi*) and depletion of candidates. Genotypes tested were (d) positive control *PASK mad2*^{RNAi} (e) negative control *mCherry mad2*^{RNAi} (f) *ADSS*^{RNAi} *mad2*^{RNAi} (g) *PRPS2*^{RNAi} *mad2*^{RNAi} (h) *TKL*^{RNAi} *mad2*^{RNAi}.

Biology Open • Supplementary information

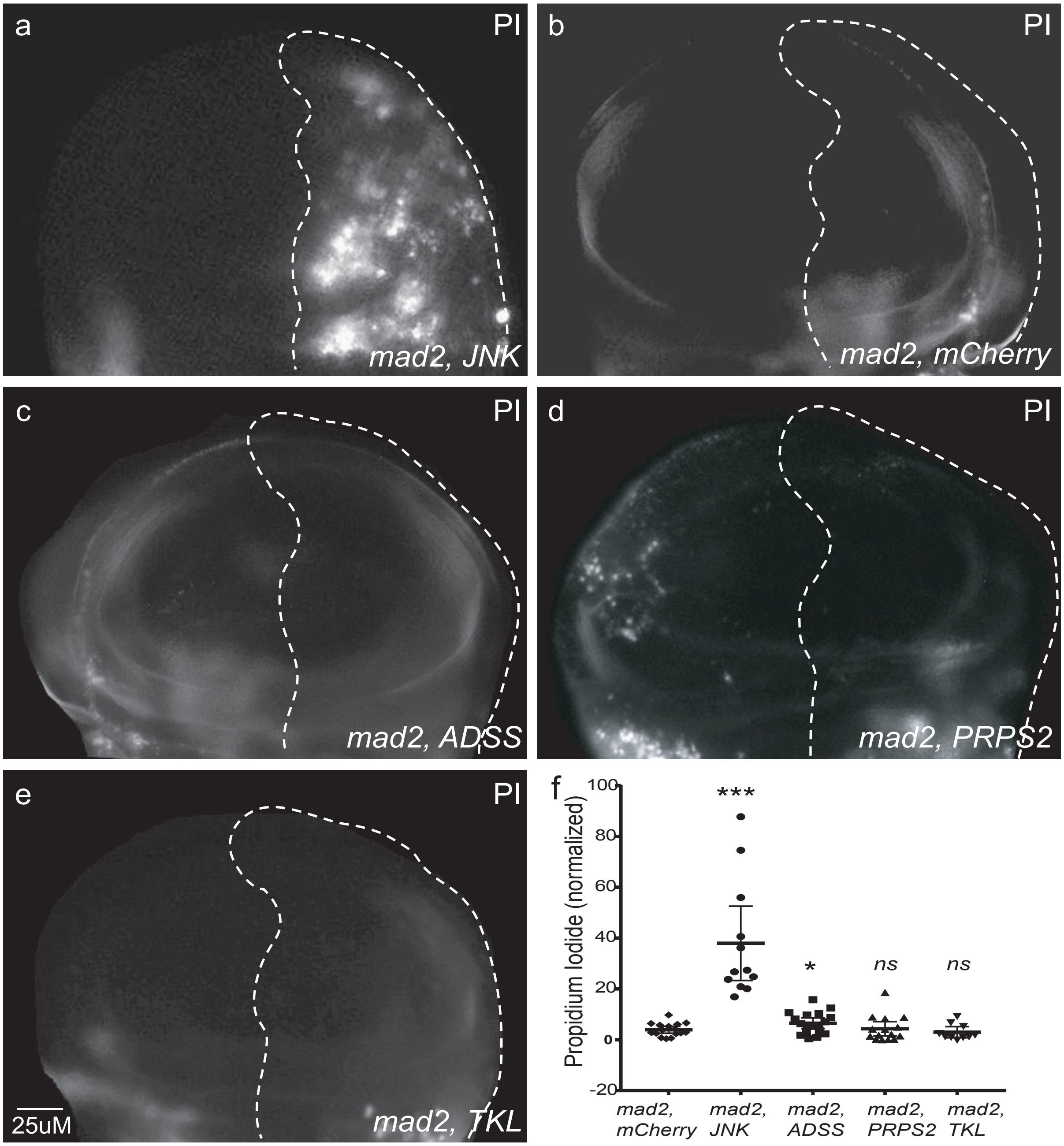

Figure S2: Propidium Iodide (PI) staining was used to measure the level of necrosis in nucleotide depleted CIN cells. The candidate knockdowns in the posterior half of each wing disc are indicated by the dotted line; the remainder of each disc was wild type. PI staining was high in positive control (a) *JNK*^{RNAi}*mad2*^{RNAi}, while (b) negative control *mCherry mad2*^{RNAi}, and the other genotypes (c) *ADSS*^{RNAi}*mad2*^{RNAi}, (d) *PRPS2*^{RNAi}*mad2*^{RNAi}, (e) *TKL*^{RNAi}*mad2*^{RNAi} were negative for PI staining. (f) Quantification of PI staining. The y axis shows the normalized PI staining obtained by the subtracting the mean value of the control region from the mean of the affected region for each wing disc. The error bars indicate the 95% confidence interval, n≥10 in all cases. The p values were calculated by two-tailed t-tests with Welch's correction. $p < 0.001$= ***.

Biology Open • Supplementary information

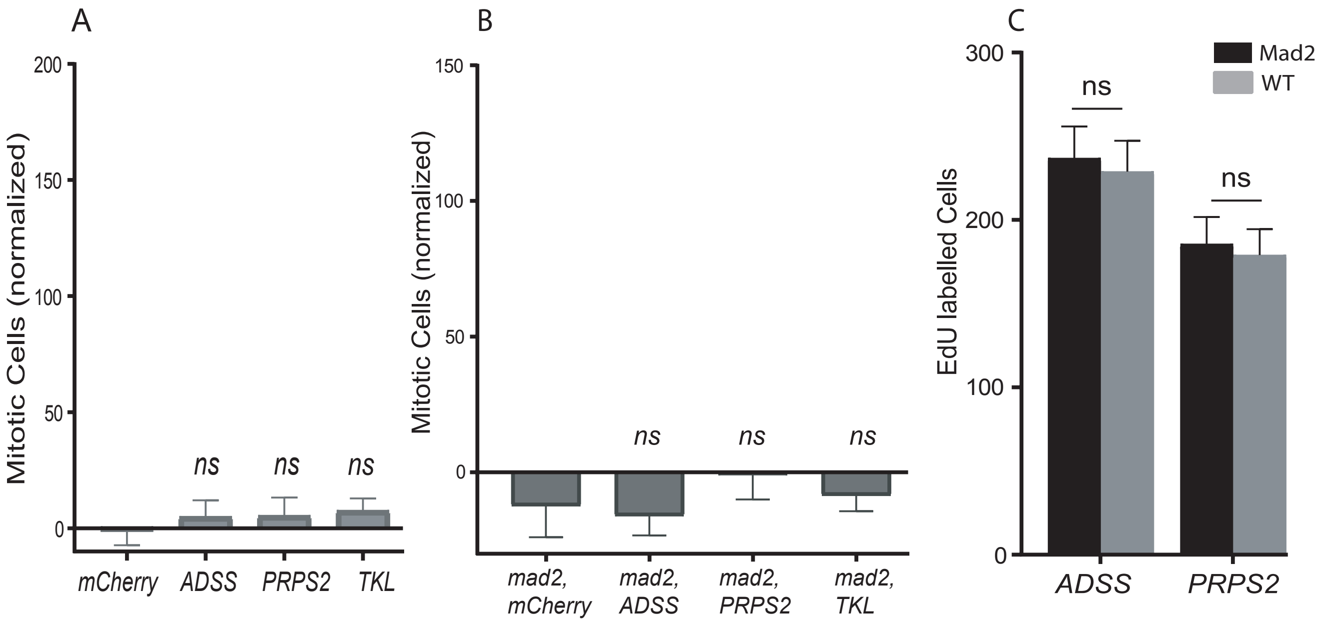

Figure S3: The effect of nucleotide depletion on the cell cycle in CIN cells. Phospho-Histone 3 antibody staining was used to detect the mitotic cells in CIN larval wing discs with nucleotide candidate knockdowns. (**A**) no significant difference was observed in the frequency of mitotic cells in wing discs of control and genotype $ADSS^{RNAi}$, $PRPS2^{RNAi}$ and TKL^{RNAi} when these candidates depleted with or without CIN as shown in graph (**B**). The error bar represents the 95% CIs, n≥8 in all cases. The p values were calculated by two-tailed t-tests with Welch's correction. (**C**) Quantification of EdU labelling on nucleotide synthesis deficient CIN cells. EdU labelling was used to detect the S-Phase cells in CIN larval wing discs with nucleotide candidate knockdowns. No significant difference was observed in the frequency of S-Phase cells in wing discs of control and genotype $\textit{ADSS}^{RNAi}$ and $\textit{PRPS2}^{RNAi}$ when these candidates depleted with or without CIN as shown in graph.

Biology Open • Supplementary information

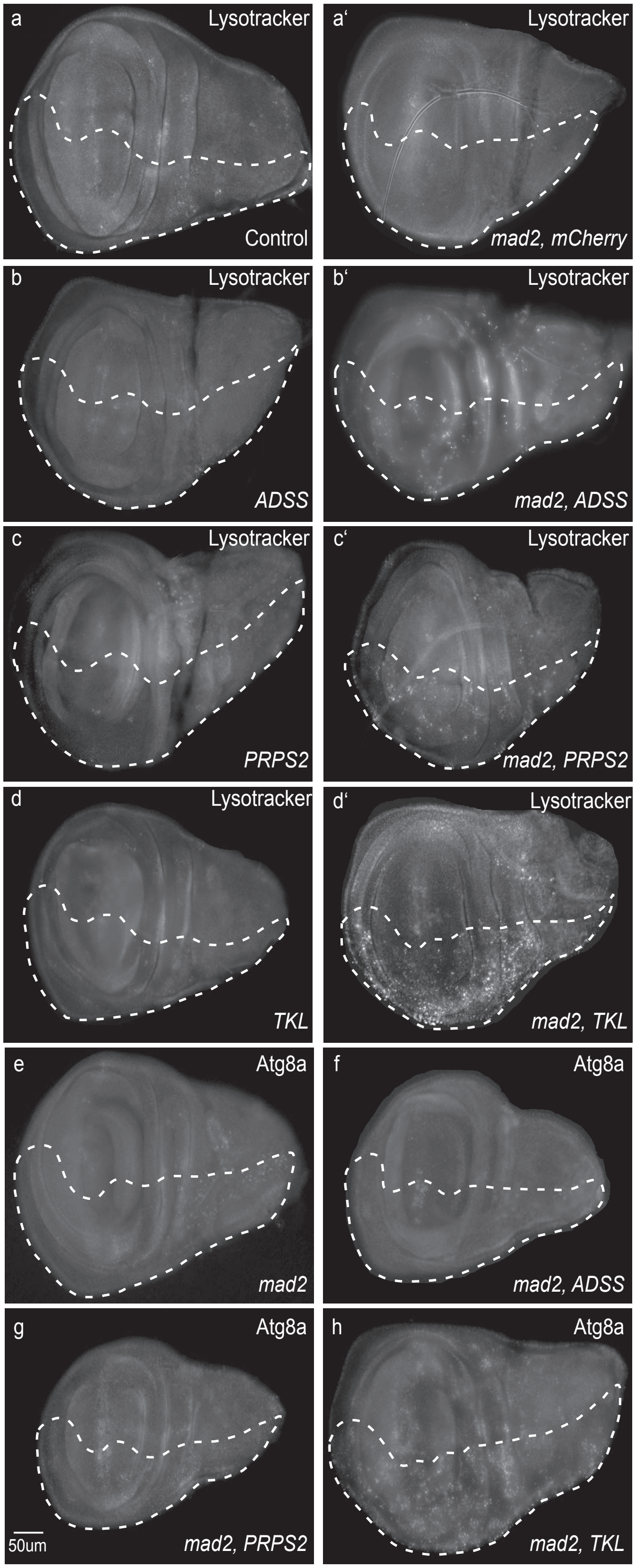

Figure S4: Third instar larvae wing discs were stained with lysotracker and mCherry-Atg8a puncta level detection to test for effects on lysosomes. In every disc, the unmarked region does not express RNAi constructs, while the dashed line shows the area affected by CIN (*mad2-RNAi*) and depletion of nucleotide synthesis enzymes. Control wings (a, a') show no lysotracker staining when CIN is induced. (b, c, d) Imaginal discs in which candidates have been depleted giving rise to no lysotracker staining. (b', c', d') Imaginal discs showing that when candidates were depleted in CIN cells high lysotracker was observed compared to controls. (e) negative control *UAS-mCherry-Atg8 UAS-mad2^{RNAi}* (f, g) Wing discs of *ADSS*^{RNAi} and *PRPS2*^{RNAi} with CIN induced by mad2 depletion (*engrailed* > *Gal4*, *UAS-CD8-GFP, UAS-mCherry-Atg8 UAS-mad2^{RNAi}*) show no induction of autophagy (mCherry-Atg8a puncta). (h) Depletion of TKL in CIN background shows a high level of mCherry-Atg8a puncta, indicating the activation of autophagy

Biology Open • Supplementary information

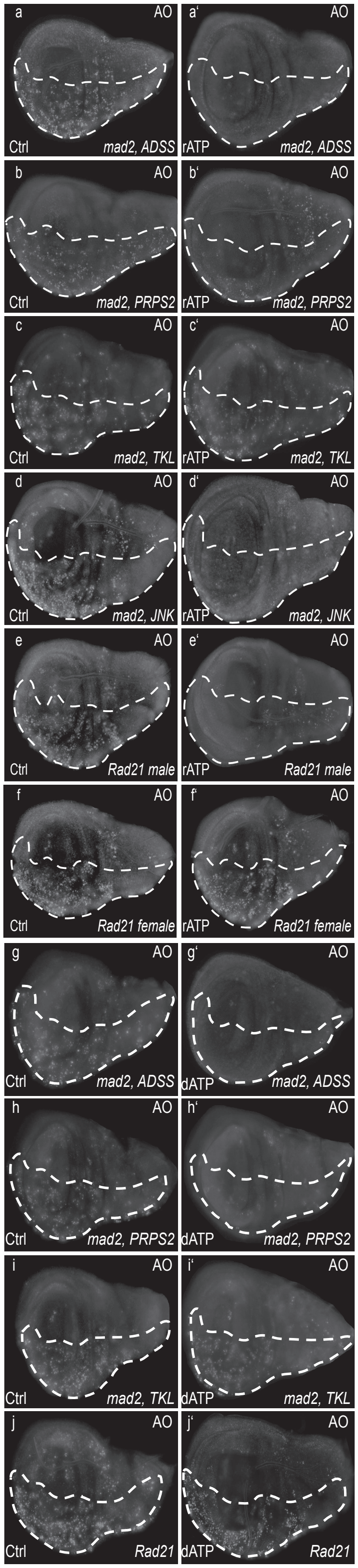

Figure S5: Depletion of nucleotide synthesis enzymes in CIN cells showed high AO staining which was rescued by feeding the larvae with rATP and dATP (1mM). (a, b, c, d) controls wing disc of candidates $ADSS^{RNAi}$, $PRPS2^{RNAi}$, TKL^{RNAi} and JNK^{RNAi} depleted in CIN cells induced by $mad2^{RNAi}$. (a', b', d') wing discs of rATP fed larvae of $ADSS^{RNAi}mad2^{RNAi}$, $PRPS2^{RNAi}mad2^{RNAi}$ and $JNK^{RNAi}mad2^{RNAi}$ which show reduction in the AO phenotype. (c') no rescue was observed in ATP fed larvae of genotype $TKL^{RNAi}mad2^{RNAi}$. (e, f) control wing discs of male and female larvae with high CIN induced by Rad21 depletion (UAS-$Rad21^{RNAi}$, UAS-*Dicer2*) show a high AO phenotype but (e') feeding rATP to male larvae of genotype (UAS-$Rad21^{RNAi}$, UAS-*Dicer2*) rescues the AO phenotype in wing discs while (f') female larvae of same genotype did not show a reduction in the AO phenotype. Feeding larvae with dATP (1mM) rescued the AO phenotype in ADSS and PRPS2 knockdowns in CIN cells (g', h') as compared to their controls (g, h). No rescue was observed in dATP fed larvae of genotypes $TKL^{RNAi}mad2^{RNAi}$ and (UAS-$Rad21^{RNAi}$, UAS-*Dicer2*) (i', j') as compared to their controls (i, j).

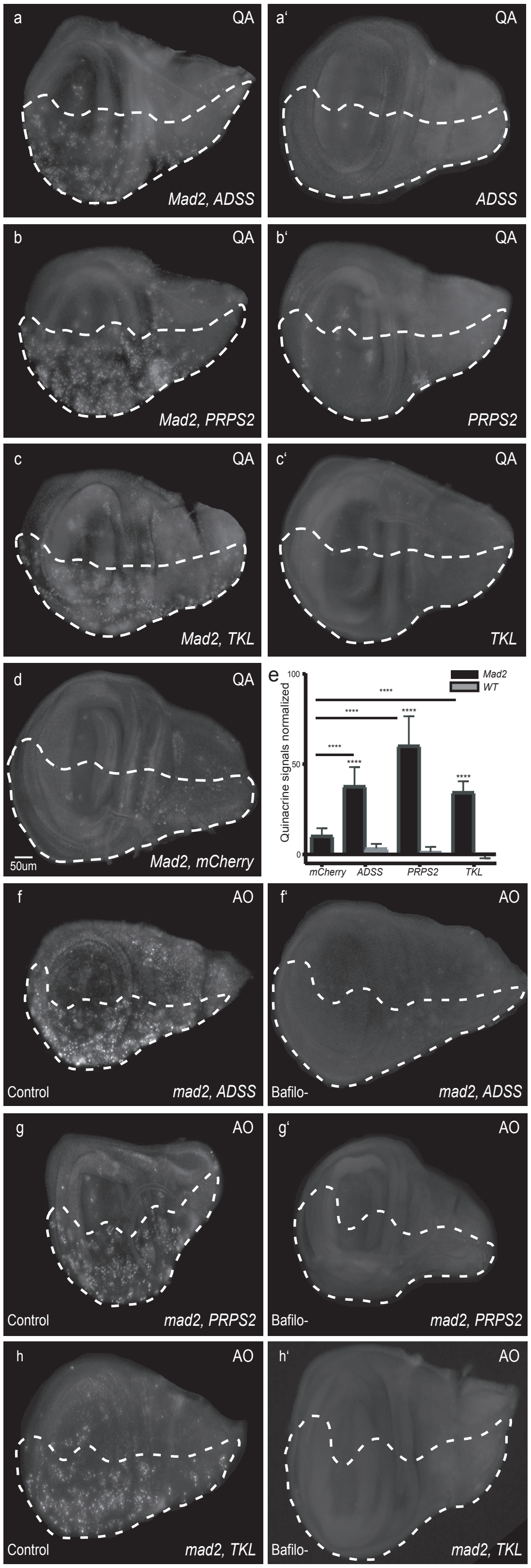

Figure S6: Effect of quinacrine (QA) staining on larval wing discs with depletion of nucleotide synthesis enzymes in CIN cells. All wing discs were stained with QA (50uM). The dotted line shows the *en*>CD8GFP marked posterior compartment in which the genes were depleted. The other half of each disc serves as a wild type internal control. (a, b, c,) high QA staining was observed, when nucleotide candidates ADSS, PRPS2 and TKL were depleted in CIN cells as compared to candidates knocked down in non-CIN cells (a', b', c'). (d) Control disc of *mCherry mad2*$^{\mathrm{RNAi.}}$ (e) Quantification of QA stainings show the normalized grey value of staining, obtained by subtracting the mean grey value of the wild type from the affected region of each disc. The error bars represent the 95% CIs, n ≥10 in all cases. The p values were calculated by two-tailed t-tests with Welch's correction. (f, g, h) control wing discs of nucleotide synthesis enzymes ADSS, PRPS2 and TKL depleted in CIN cells show high AO phenotypes. (f', g', h') wing discs of same genotype treated with bafilomycin (75nM) treatment for 30 min significantly reduced the AO staining.

Biology Open • Supplementary information

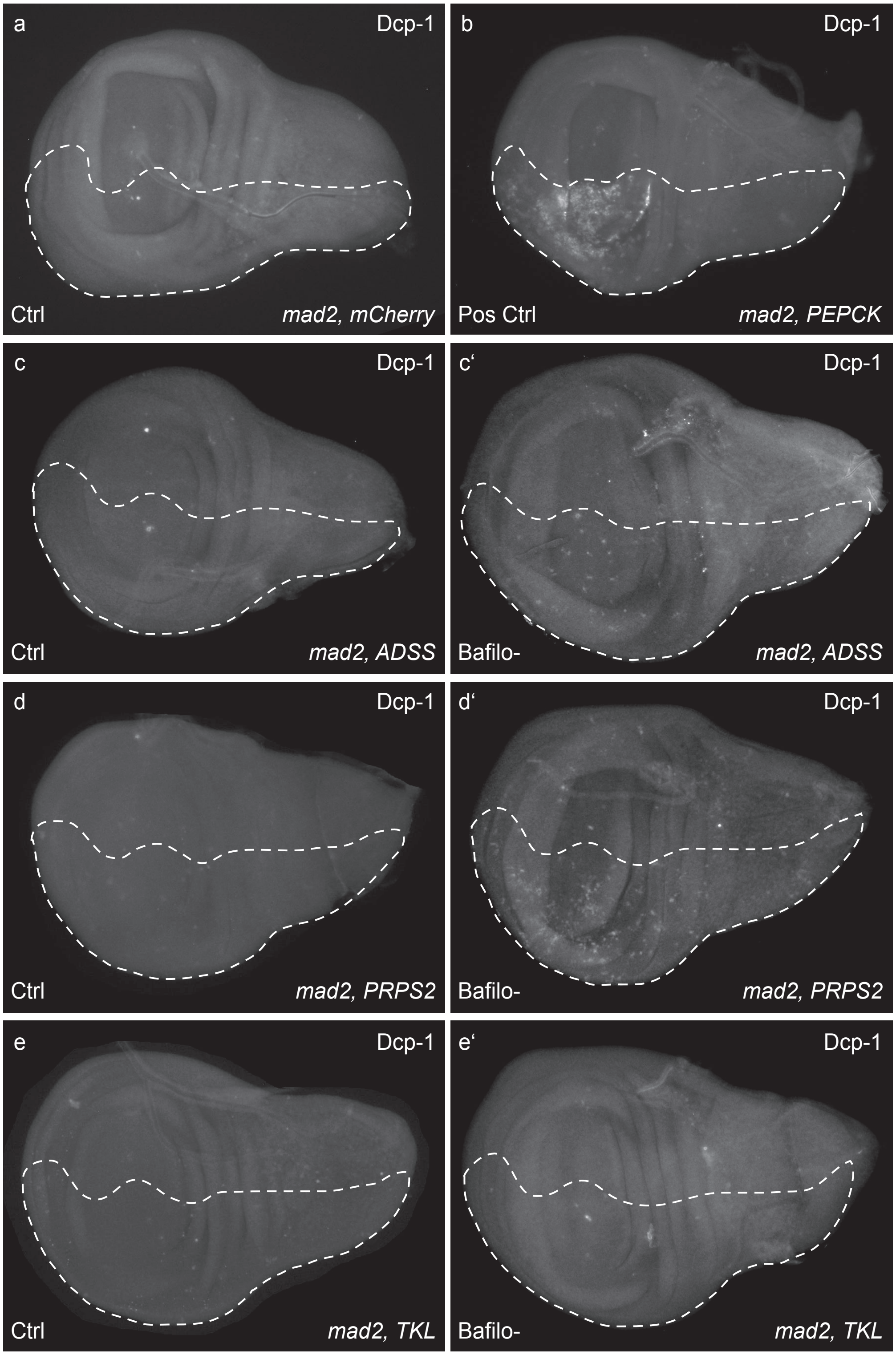

Figure S7: Blocking v-ATPase activity causes some apoptosis in nucleotide depleted CIN cells. Dcp-1 antibody staining was used to detect the level of apoptosis in bafilomycin treated wing discs of candidates' knockdowns in CIN cells. (a) Negative control (b) positive control *PEPCK*^{RNAi} *mad2*^{RNAi}. (c, d, e) wing discs of knockdowns of ADSS, PRPS2 and TKL in CIN cells without bafilomycin treatment show no apoptosis. (c', d') Knockdown of ADSS and PRPS2 significantly increased the level of apoptosis in CIN cells after treatment with bafilomycin. (e') knockdown of TKL in CIN cells did not show significant apoptosis in these proliferating cells.

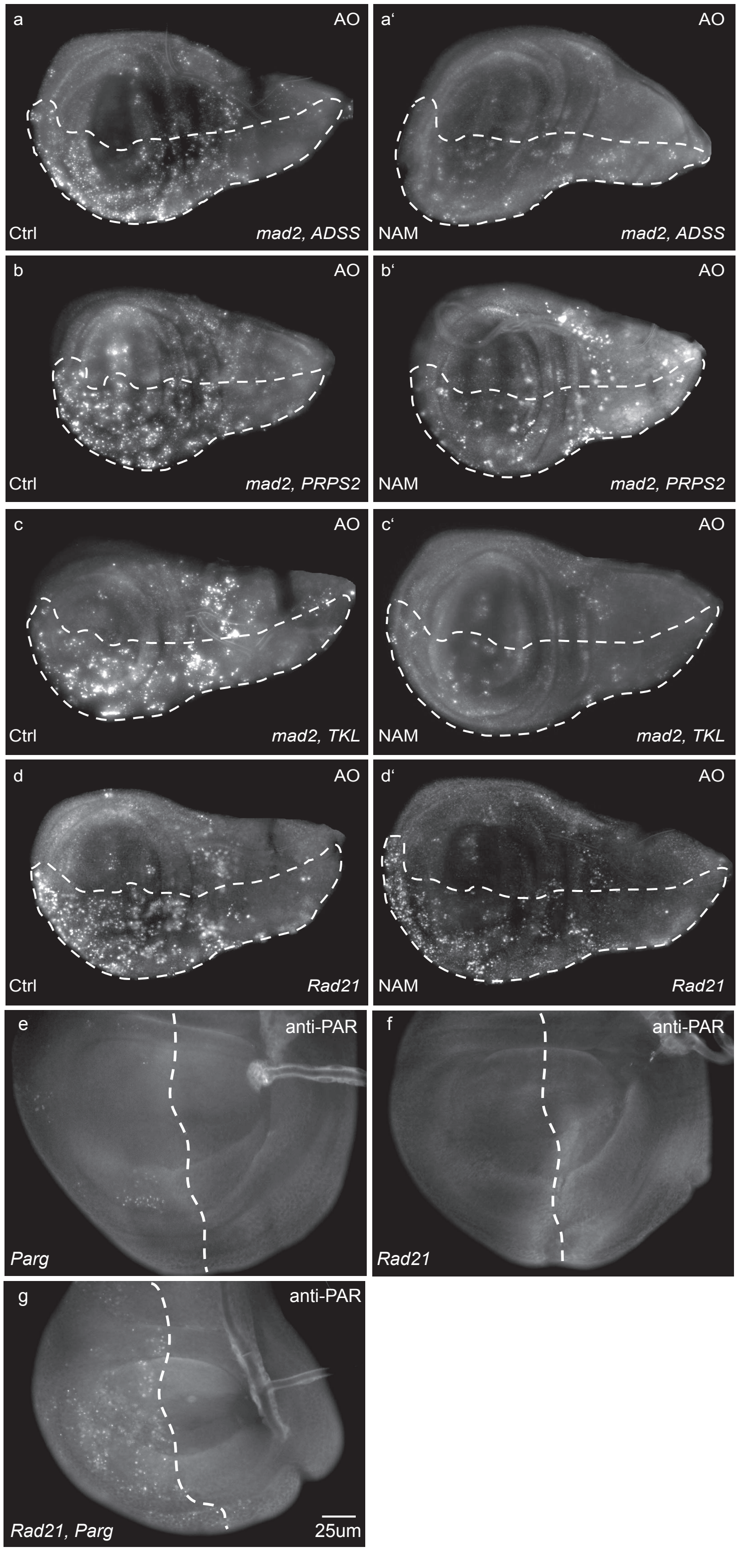

Figure S8: Nicotinamide rescues the AO phenotype in CIN cells. (a, b, c) represent the AO staining in control wing discs without NAM treatment of candidates *ADSS*^{RNAi}, *PRPS2*^{RNAi} and *TKL*^{RNAi} in non-CIN cells. (a', b', c',) feeding the larvae with NAM (1mM) significantly rescued the AO in wing disc of genotype *ADSS*^{RNAi}, *PRPS2*^{RNAi} and *TKL*^{RNAi} in CIN cells. (d) Wing discs with high CIN induced by Rad21 depletion showed high AO staining. (d') feeding NAM to the larvae with high CIN caused reduced AO staining. (g) increased in anti-PAR antibody staining in Parg with high-CIN cells (UAS-*Rad21*^{RNAi}, UAS-*Dicer2*) was observed compared to the controls alone (e, f).

Biology Open • Supplementary information
